# Iturin derived from *Bacillus* modulates lipid and glucose metabolism while mitigating the progression of MASLD

**DOI:** 10.1016/j.isci.2026.115470

**Published:** 2026-03-25

**Authors:** Lingyun Zhao, Qing Liu, Jianuo He, Yujian Li, Yang Zhang, Yingxue Feng, Wenya Zhao, Liping Zhang, Yongfeng Liu, Tongliang Li, Hongwei Liu

**Affiliations:** 1 Institute of Biology, Hebei Academy of Sciences, Shijiazhuang, Hebei 050011, China; 2 Department of Radiation Oncology, Henan Provincial Key Laboratory of Radiation Medicine, The First Affiliated Hospital of Zhengzhou University, Zhengzhou, Henan 450052, China

**Keywords:** hepatology

## Abstract

Metabolic dysfunction-associated steatotic liver disease (MASLD), a metabolic disorder associated with modern dietary patterns, urgently requires safe dietary interventions for prevention. This study explored the bioactivity of iturin (IT), a *Bacillus*-derived lipopeptide, in modulating hepatic lipid/glucose metabolism and alleviating MASLD. IT supplementation ameliorated MASLD and metabolic dysregulation in high-fat diet-fed mice, evidenced by reduced body weight, attenuated hepatic lipid accumulation, and improved glucose homeostasis. Concurrently, IT altered gut microbiota composition and modified bile acid metabolism profiles. At the mechanistic level, decreased hepatic Cd36 expression was observed in mice receiving IT supplementation. These findings highlight IT as a dietary bioactive compound targeting lipid and glucose metabolism, offering a potential strategy for mitigating MASLD progression through functional food or nutraceutical applications.

## Highlights

• Iturin (IT) alleviates high-fat-diet-induced MASLD and reduces body weight
• IT demonstrates regulatory effects on glycolipid metabolic processes in mice
• IT demonstrates inhibitory effects on *Cd36* expression
• IT remodels the gut microbiota and regulates bile acid metabolism in mice

## Graphical abstract

**Figure:**
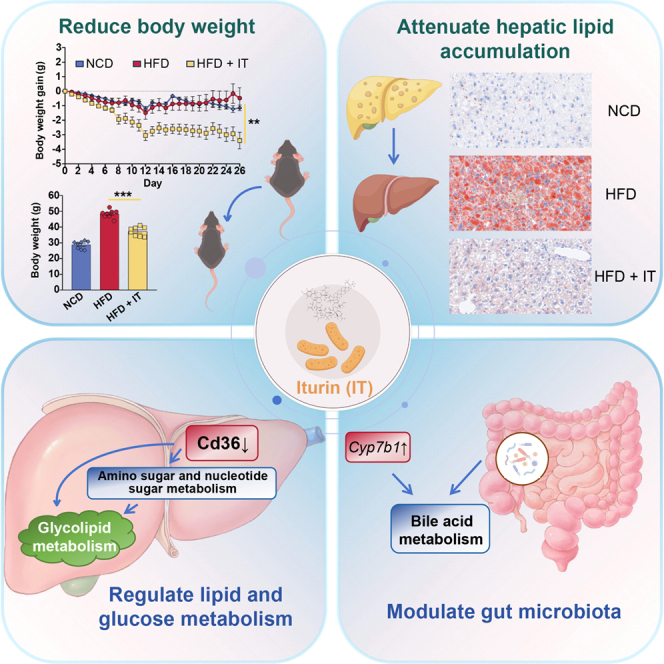

## Introduction

Metabolic dysfunction-associated steatotic liver disease (MASLD), the most prevalent chronic liver disorder, affects over 32% of the global adult population and poses a growing socioeconomic burden due to its association with obesity and metabolic syndrome.^1^^,^^2^^,^^3^ Progressive MASLD manifests as triglyceride (TG) accumulation (steatosis), advancing to metabolic dysfunction-associated steatohepatitis (MASH) and hepatocellular carcinoma in 10–20% of cases.^4^

Despite its pandemic prevalence, clinically approved pharmacological interventions remain extremely scarce, with lifestyle interventions remaining the primary management strategy.^5^ The recent accelerated approval of resmetirom (Rezdiffra) for MASH^6^ and several other agents (e.g., FXR/PPAR agonists)^7^ in clinical development highlight the ongoing unmet need for effective therapies. In the absence of specific pharmacotherapies, certain guidelines have suggested medications such as vitamin E and Pioglitazone to address metabolic disturbances, though these do not directly target MASH pathology.^8^ This gap underscores the urgency to identify safe, food-compatible bioactive agents capable of modulating key metabolic pathways in MASLD.

Emerging evidence implicates hepatic nutrient oversupply, particularly excessive free fatty acids (FFAs) and glucose influx, as a critical driver of MASLD progression. The “multiple parallel-hit” model emphasizes synchronized dysregulation in lipid/glucose homeostasis, mitochondrial dysfunction, and oxidative stress.^9^ Targeting hub regulators governing systemic metabolism (e.g., fatty acid transporters such as *Cd36*) may offer broader therapeutic efficacy than single-pathway inhibition. Diet-derived compounds with pleiotropic metabolic effects, such as probiotics^10^ and polyphenols,^11^^,^^12^^,^^13^ are gaining attention as natural alternatives, demonstrating the potential of multi-target nutritional interventions.^14^

Bioactive peptides from food-grade microorganisms represent an underexplored resource for MASLD intervention. Cyclic lipopeptides (CLPs) from *Bacillus* spp., widely employed as food preservatives and biocontrol agents,^15^ demonstrate metabolic modulatory potential beyond their antimicrobial properties. Iturin (IT), a *Bacillus subtilis*-derived CLP, exhibits antifungal and antitumor activities,^16^ yet its effects on hepatic metabolism remain unknown. Herein, we characterize IT as a MASLD-alleviating agent through an integrated *in vivo* and multi-omics approach. Our findings reveal IT’s capacity to curb diet-induced weight gain, mitigate hepatic steatosis, and regulate fatty acid/glucose metabolism. By positioning IT within the context of food-based metabolic therapeutics, this work bridges microbial biotechnology and nutritional hepatology, offering insights into sustainable strategies for MASLD management.

## Results

### Iturin supplementation attenuates high-fat diet-induced body weight gain in mice

The food-grade lipopeptide IT, obtained through ultrafiltration coupled with preparative HPLC (Figures S1 and S2), was evaluated for its protective effects against hepatic steatosis in high-fat diet (HFD)-fed mice. Prior to long-term intervention, a preliminary pharmacokinetic (PK) study was performed in mice fed a normal diet. Following a single oral administration of IT (14 mg/kg, prepared at 2.1 mg/mL with an adjusted volume of approximately 200 μL per mouse based on body weight), tissue distribution was analyzed at 2 h post-dose. The results showed that IT was predominantly distributed in the feces (intestinal content), exhibiting the highest relative level (10.10% relative to the reference standard, which was identical in concentration to the administered solution at 2.1 mg/mL). This was followed by blood (4.06%). Moderate distribution was observed in the small intestine (0.57%), colon (0.24%), liver (0.23%), spleen (0.21%), and kidney (0.19%), whereas lower levels were detected in the heart (0.11%), brain (0.10%), and lung (0.06%) (Figure S3). These findings collectively supported the rationale for subsequent oral therapeutic intervention.

**Figure 1:**
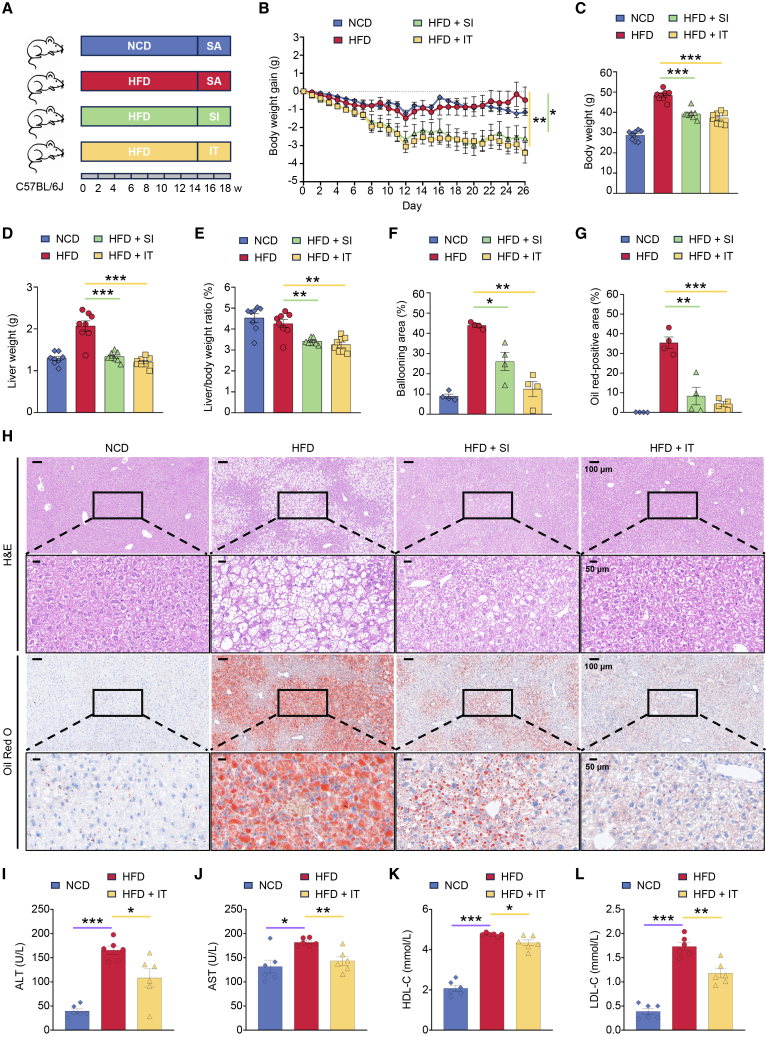
Iturin supplementation alleviates hepatic steatosis and improves serum biomarkers in HFD-fed mice (A) Schematic of the experimental design. C57BL/6J mice (*n* = 8 per group) were fed a normal chow diet (NCD) or a high-fat diet (HFD) for 18 weeks. After an initial 14-week modeling period, HFD-fed mice received daily oral gavage of saline (SA, vehicle), silybin (SI, 57 mg/kg), or iturin (IT, 14 mg/kg) for the following 4 weeks. NCD-fed mice received daily gavage of saline as a control during the same treatment period. (B) Body weight gain of mice (*n* = 8 per group). Data are presented as mean ± SEM. Statistical significance was determined by one-way ANOVA with Bonferroni post hoc correction. ∗*p* < 0.05 and ∗∗*p* < 0.01. (C–E) The body weight (C), liver weight (D), and liver/body weight ratio (E) of mice on the final day of the experiment (*n* = 8 per group). Data are presented as mean ± SEM. Statistical significance was determined by unpaired Student’s *t* test, comparing the treatment group (HFD + SI, HFD + IT) with the HFD group. ∗∗*p* < 0.01 and ∗∗∗*p* < 0.001. (F and G) Quantitative analysis of the ballooning area of the liver (F) and Oil red O-positive area of the liver (G) (*n* = 4 per group). Data are presented as mean ± SEM. Statistical significance was determined by unpaired Student’s *t* test, comparing the treatment group (HFD + SI, HFD + IT) with the HFD group. ∗*p* < 0.05, ∗∗*p* < 0.01, and ∗∗∗*p* < 0.001. (H) The representative images of liver sections stained with H&E and Oil red O. (Low-magnification view, scale bars, 100 μm; High-magnification view, scale bars, 50 μm). (I–L) Serum levels of ALT (I), AST (J), HDL-C (K), and LDL-C (L) at the end of the experiment (*n* = 6 per group). Data are presented as mean ± SEM. Statistical significance was determined by unpaired Student’s *t* test, comparing the HFD group with the NCD group, and the HFD + IT group with the HFD group. ∗*p* < 0.05, ∗∗*p* < 0.01, and ∗∗∗*p* < 0.001.

Silybin (SI), a hepatoprotective phytochemical sourced from *Silybum marianum* (milk thistle) extract with established antioxidant and anti-inflammatory activity in functional food applications, was utilized as the reference compound for efficacy benchmarking.^17^ After establishing metabolic dysregulation through 14-week HFD feeding, mice sequentially underwent a 4-week therapeutic dietary intervention protocol involving daily oral gavage administration of three regimens: 0.9% saline (SA) (HFD controls); 57 mg/kg/day SI (10× the typical food additive dosage) (HFD + SI); 14 mg/kg/day IT (HFD + IT). Control cohorts maintained on a normal-caloric diet (NCD) received daily oral administration of 0.9% SA as an iso-caloric vehicle control (NCD controls), ensuring parity in fluid intake volume compared to experimental dietary interventions (Figure 1A). Critically, the respective NCD or HFD dietary regimens were maintained unchanged for all groups throughout the entire 4-week intervention period.

Initiation of therapeutic intervention elicited divergent body weight dynamics across experimental groups. The transient, minor weight reduction observed across all groups during the initial days of gavage was likely attributable to adaptive metabolic responses to the daily handling and administration procedure. Both NCD and HFD control cohorts exhibited an adaptive weight stabilization phase during the initial 12 days (<1.5 g absolute change), whereafter HFD-fed mice resumed progressive weight gain culminating in significantly elevated terminal mass (48.4 ± 0.9 g at day 26, *p* < 0.001 vs. NCD: 28.7 ± 0.9 g). In contrast, mice receiving SI and IT via daily gavage maintained progressive weight reduction throughout the intervention period. By endpoint (day 26), IT-supplemented attained a mean body weight of 37.2 ± 1.0 g (*p* < 0.001 vs. HFD controls) with a net reduction of 3.4 ± 0.6 g (*p* < 0.01 vs. HFD controls), while SI-supplemented counterparts reached 39.2 ± 0.9 g (*p* < 0.001 vs. HFD controls) with a 2.6 ± 0.6 g (*p* < 0.05 vs. HFD controls) reduction (Figures 1B and 1C). This sustained negative energy balance establishes IT as an effective agent for reversing diet-induced weight gain.

### Iturin attenuates high-fat diet-induced hepatic lipid accumulation and ameliorates MASLD in mice

Concurrent with the observed body weight modulation, both IT and SI supplementation significantly ameliorated hepatic enlargement induced by the HFD regimen. At terminal necropsy, the physiological reference established by NCD-fed mice demonstrated liver parameters at 1.29 ± 0.05 g and adiposity-unadjusted hepatosomatic indices of 4.53 ± 0.21%. In sharp contrast, HFD controls developed significant hepatic pathology, manifesting absolute liver mass at 2.07 ± 0.12 g and indices of 4.26 ± 0.20%. Critically, both IT and SI interventions attenuated these diet-induced abnormalities. IT supplementation yielded liver weight reduction to 1.21 ± 0.04 g (*p* < 0.001 vs. HFD controls) with indices reduced to 3.25 ± 0.13% (*p* < 0.01 vs. HFD controls), while SI supplementation achieved 1.34 ± 0.04 g (*p* < 0.001 vs. HFD controls) and 3.41 ± 0.05% (*p* < 0.01 vs. HFD controls), respectively (Figures 1D and 1E). The concordant reduction in both absolute and relative liver metrics underscores the hepatoprotective efficacy of IT against diet-induced steatosis.

Four mice per group were randomly selected for liver histopathological examination. Histological analyses via hematoxylin and eosin (H&E) staining demonstrated preserved hepatocyte architecture in NCD-fed mice, whereas HFD-fed mice exhibited severe hepatic ballooning, with ballooning areas occupying 43.86 ± 0.78% of hepatic tissue sections. Crucially, this pathological alteration was markedly attenuated by both IT and SI supplementation, which reduced ballooning areas to 12.46 ± 3.75% (*p* < 0.01 vs. HFD controls) and 26.02 ± 4.45% (*p* < 0.05 vs. HFD controls), respectively. Consistent with these findings, Oil Red O staining revealed extensive lipid accumulation in HFD liver specimens (positive area: 35.37 ± 2.96%), which was significantly ameliorated by IT (4.34 ± 1.31%, *p* < 0.001) and SI supplementation (8.35 ± 4.49%, *p* < 0.01), indicating the potential of IT in ameliorating MASLD induced by a HFD in mice (Figures 1F–1H).

To further evaluate hepatic function and systemic metabolic status, serum biochemical parameters were assessed (Figures 1I–1L). HFD feeding induced significant liver injury and dyslipidemia, as evidenced by markedly elevated levels of alanine aminotransferase (ALT), aspartate aminotransferase (AST), high-density lipoprotein cholesterol (HDL-C), and low-density lipoprotein cholesterol (LDL-C) compared to the NCD group. Supplementation with IT significantly counteracted these HFD-induced changes, leading to a pronounced reduction in ALT, AST, and LDL-C levels. A decreasing trend was also observed in HDL-C levels following IT treatment, although the magnitude of change was less dramatic.

To assess systemic glucose metabolism, an oral glucose tolerance test (OGTT) was performed. Mice fed an HFD exhibited significantly elevated blood glucose levels compared to NCD-fed mice throughout the test period. Although the baseline fasting blood glucose in IT-supplemented HFD mice was comparable to that of the HFD control group, a non-significant trend toward improved glucose tolerance was observed following glucose challenge (Figure S4).

**Figure 2:**
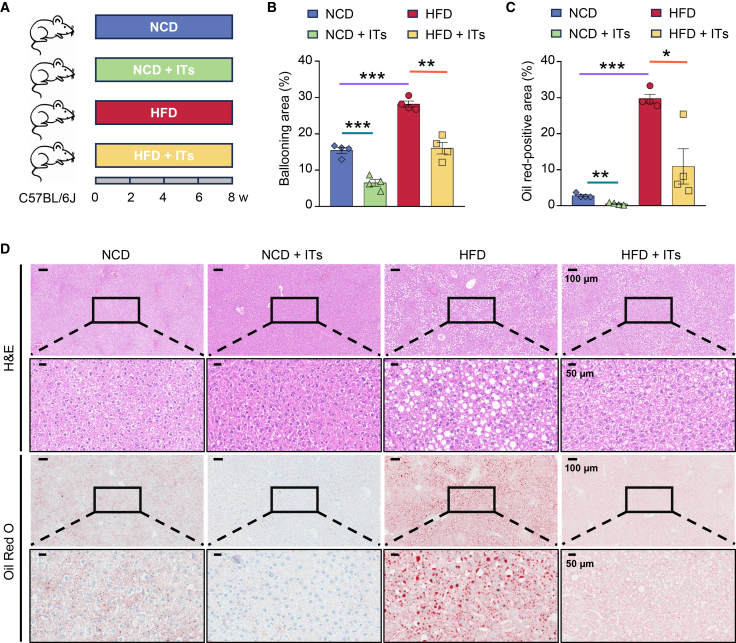
Iturin protects against diet-induced liver injury and steatosis (A) Schematic of the experimental design. C57BL/6J mice (*n* = 6 per group) were randomly allocated into four groups and treated for 8 weeks. Mice fed an NCD received either regular water or water containing iturin (ITs, 0.1 g/L), while mice fed an HFD received either regular water or iturin-supplemented water. (B and C) Quantitative analysis of the ratio of ballooning area (B) and the ratio of Oil red O-positive area (C) of the liver (*n* = 4 per group). Data are presented as mean ± SEM. Statistical significance was determined by unpaired Student’s *t* test, comparing the HFD group with the NCD group, the NCD + IT group with the NCD group, and the HFD + IT group with the HFD group. ∗*p* < 0.05, ∗∗*p* < 0.01, and ∗∗∗*p* < 0.001. (D) Representative images of H&E staining and Oil red O staining of liver sections. (Low-magnification view, scale bars, 100 μm; High-magnification view, scale bars, 50 μm).

To evaluate the efficacy of IT as a functional food additive in mitigating MASLD, supplemental IT (0.1 g/L in drinking water; equivalent to 14 mg/kg/day) was administered to mice fed either NCD (NCD + ITs) or HFD (HFD + ITs), with respective control cohorts maintained on drinking water without additives over an 8-week period (Figure 2A). H&E staining quantification demonstrated significantly attenuated hepatic ballooning in IT-supplemented groups (NCD + ITs: 6.51 ± 0.93% vs. NCD: 15.46 ± 0.85%, *p* < 0.001; HFD + ITs: 16.06 ± 1.61% vs. HFD: 28.19 ± 0.84%, *p* < 0.01) (Figures 2B and 2D). Concordantly, oil red O staining revealed reduced lipid-positive areas (NCD + ITs: 0.54 ± 0.17% vs. NCD: 2.80 ± 0.30%, *p* < 0.01; HFD + ITs: 10.94 ± 4.90% vs. HFD: 29.72 ± 1.23%, *p* < 0.05) (Figures 2C and 2D), collectively indicating potent hepatoprotection by IT supplementation. Collectively, these findings demonstrate that the *Bacillus*-derived IT exhibits hepatic protective effects, highlighting its applicability in diet-based attenuation strategies for MASLD progression.

### Iturin influences glycolipid metabolism and redox-related metabolic processes in both human and murine systems

**Figure 3:**
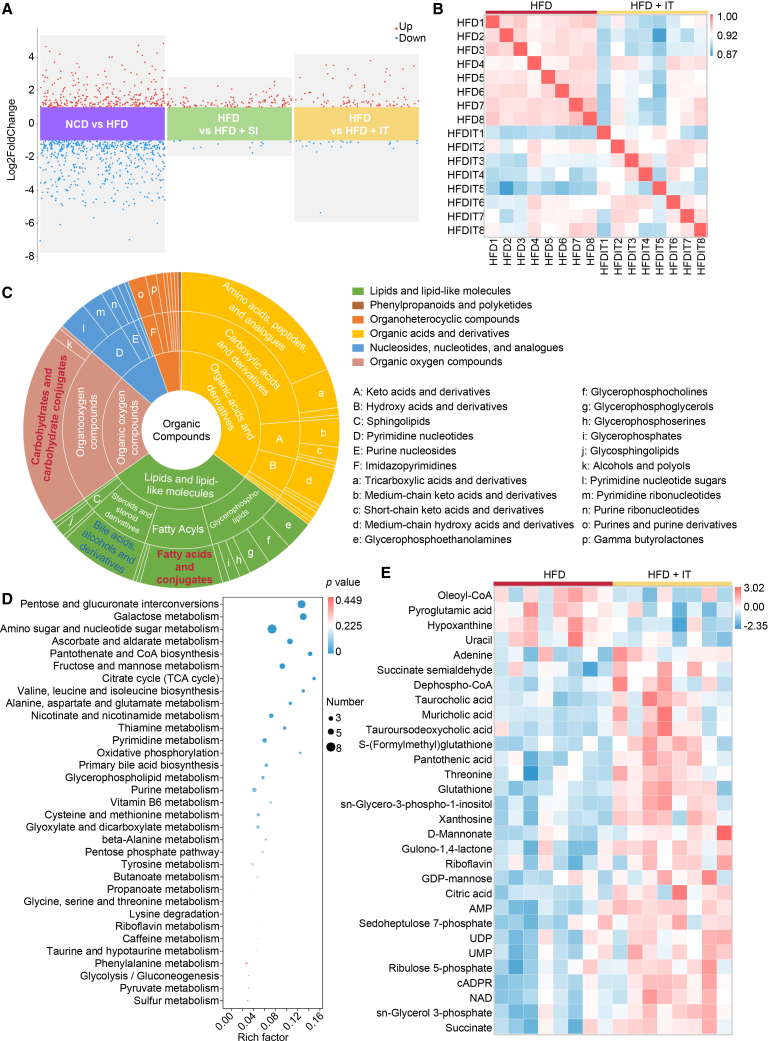
Hepatic metabolomic profiling reveals iturin-induced alterations in HFD-fed mice All data presented herein were generated from samples collected in the first experimental series/therapeutic model. (A) Volcanic plot depicts variations in metabolite profiles (*n* = 8 per group). Metabolites with |log_2_ fold change| > 1 and adjusted *p* value <0.05 (by Mann-Whitney U test with Benjamini-Hochberg correction) are highlighted in red (up-regulated) and blue (down-regulated). (B) Correlations among metabolites within liver samples of HFD-fed mice treated with either saline or iturin (*n* = 8 per group). (C) Pie charts show the classification of differential metabolites identified between the HFD and HFD + IT. (D) KEGG pathway enrichment analysis of differentially expressed metabolites between HFD-fed mice treated with saline and those treated with iturin. Bubble size represents the number of metabolites; color represents the *p* value. (E) The heatmap depicts the prominent differentially expressed metabolites in HFD-fed mice subjected to saline versus iturin treatment (*n* = 8 per group).

To elucidate the influence of IT on metabolic profiles, a metabolomics analysis was performed using liver samples from the first experiment (therapeutic intervention). The HFD led to significant alterations in liver metabolites, with 294 metabolites upregulated and 552 downregulated compared to NCD-fed mice. In contrast to HFD-fed mice receiving SA treatment, mice supplemented with IT exhibited the upregulation of 96 metabolites and downregulation of 28 metabolites (Figure 3A). IT supplementation influenced liver metabolites, demonstrating a good correlation within the HFD group but a relatively poor correlation between the HFD and IT-supplemented groups (Figure 3B). Carbohydrates and fatty acids constituted the majority of differentially expressed metabolites between groups with or without IT (Figure 3C).

Metabolic pathway enrichment analysis utilizing KEGG was employed to discern significant differences in pathways between mice treated with IT and those treated with SA (Figure 3D). The analysis revealed enrichment in various pathways, notably in carbohydrate metabolism pathways such as pentose and glucuronate interconversions, galactose metabolism, amino sugar and nucleotide sugar metabolism, and fructose and mannose metabolism. Additionally, alterations were observed in lipid metabolism pathways, particularly in glycerophospholipid metabolism. Moreover, changes were noted in pathways related to vitamin metabolism, the citrate cycle, oxidative phosphorylation, and primary bile acid biosynthesis following IT treatment.

The heat maps of differential metabolites highlighted an enrichment of bile acid-related metabolites, such as tauroursodeoxycholic acid, muricholic acid, and taurocholic acid, in mice supplemented with IT. In addition, increased levels of NAD and glutathione were observed in IT-supplemented mice. Moreover, vitamins including gulono-1, 4-lactone (an intermediate in vitamin C synthesis), pantothenic acid, and riboflavin were found to be enriched. Glycerol 3-phosphate levels were also elevated. Furthermore, UMP and UDP, associated with uridine synthesis, displayed increased levels. D-mannonate, ribulose 5-phosphate, GDP-mannose, and sedoheptulose 7-phosphate levels were also enriched, along with intermediates of the TCA cycle, such as Succinate and Citric acid (Figure 3E).

**Figure 4:**
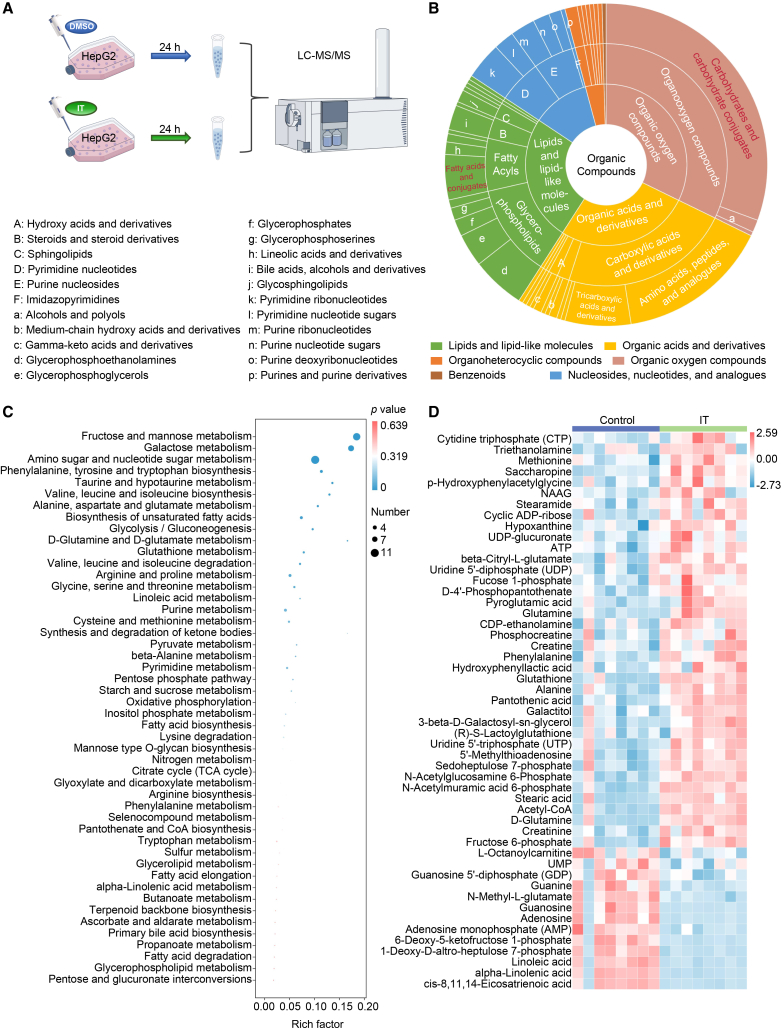
Iturin supplementation remodels the metabolome in HepG2 cells (A) The schematic representation of the HepG2 metabolomics investigation. HepG2 cells were treated with either DMSO (control) or IT (200 ng/mL) for 24 h, followed by metabolite extraction and LC-MS/MS analysis to profile the cellular metabolome (*n* = 8 per group). (B) Circular diagrams illustrate the categories of metabolites differentially abundant between IT-treated cells and controls. (C) KEGG pathway enrichment analysis of differentially expressed metabolites between IT-treated cells and controls. Bubble size represents the number of metabolites; color represents the *p* value. (D) The heatmap depicting the prominent differentially expressed metabolites, selected based on |log_2_ fold change| > 1 and adjusted *p* value <0.05 using the Mann-Whitney U test with Benjamini-Hochberg correction (*n* = 8 per group).

To assess the translational relevance of IT’s hepatic effects, *in vitro* experiments were conducted on HepG2 cells, which were supplemented with IT or DMSO for 24 h, and cell lysates were analyzed for metabolomics (Figure 4A). In alignment with the outcomes observed in mice, the administration of IT to HepG2 cells resulted in significant alterations in cellular metabolism, with notable impacts on carbohydrate metabolism, as well as some fatty acids and bile acids (Figure 4B). This was further supported by KEGG enrichment analysis, which highlighted pathways enriched in carbohydrate metabolism as well as lipid and bile acid metabolism (Figure 4C). Furthermore, IT also modulated the glutathione metabolism pathway and increased glutathione levels (Figures 4C and 4D).

Overall, IT impacted glucose metabolites, lipid metabolites, and metabolic pathways involved in glycolipid metabolism, including bile acid metabolites, among others. Additionally, IT influenced NAD metabolism and glutathione metabolism, which are associated with oxidative-reduction processes.

### Iturin modulates Cd36-dependent signaling

**Figure 5:**
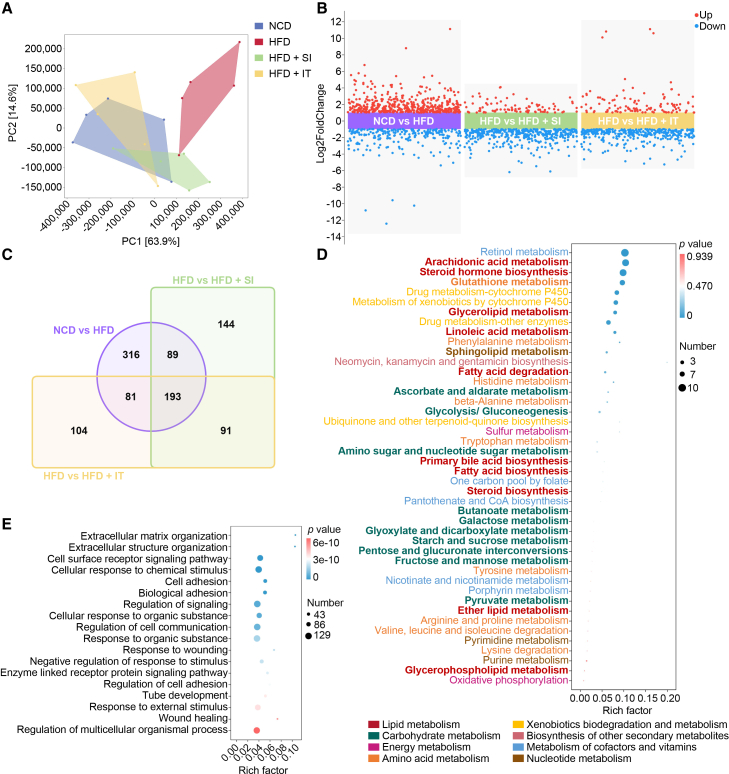
Transcriptomic analysis identifies iturin-modulated pathways in the liver All data presented herein were generated from samples collected in the first experimental series/therapeutic model. (A) Principal component analysis (PCA) of the gene expression levels in liver samples (*n* = 5 per group). (B) Volcanic plot depicts variations in DEGs (*n* = 5 per group). DEGs with |log_2_ fold change| > 1 and adjusted *p* value <0.05 (by DESeq2) are highlighted in red (up-regulated) and blue (down-regulated). (C) Venn diagram shows the overlap and unique sets of DEGs identified between different experimental conditions. (D) KEGG enrichment analysis of the metabolic-level differences in DEGs between HFD-fed mice subjected to saline and those administered with iturin. Bubble size represents the number of metabolites; color represents the *p* value. (E) GO analysis of the DEGs between mice fed HFD treated with saline and those treated with iturin. Bubble size represents the number of metabolites; color represents the *p* value.

Transcriptome analysis was conducted on mouse liver tissue from the first experiment (therapeutic intervention) to explore the mechanism of IT action. The validation of the RNAseq outcomes was carried out through qRT-PCR, affirming the reliability of the RNAseq findings (Figure S5). Liver samples underwent RNA sequencing, and principal component analysis (PCA) demonstrated distinct separation in the transcriptome profiles between NCD-fed and HFD-fed mice. Notably, a clear differentiation was observed between HFD-fed mice treated with SA and those supplemented with IT (Figure 5A). A total of 122 differentially expressed genes (DEGs) were upregulated, and 347 DEGs were downregulated in the IT-supplemented mice compared to the HFD-fed mice treated with SA (Figures 5B and 5C). Consistent with the metabolomics findings, the KEGG pathway enrichment analysis revealed alterations in carbohydrate metabolism and lipid metabolism in the IT-supplemented mice (Figure 5D). In addition, the analysis of gene ontology (GO) revealed significant alterations in the signaling pathway of cell surface receptors as a response to IT supplementation (Figure 5E).

**Figure 6:**
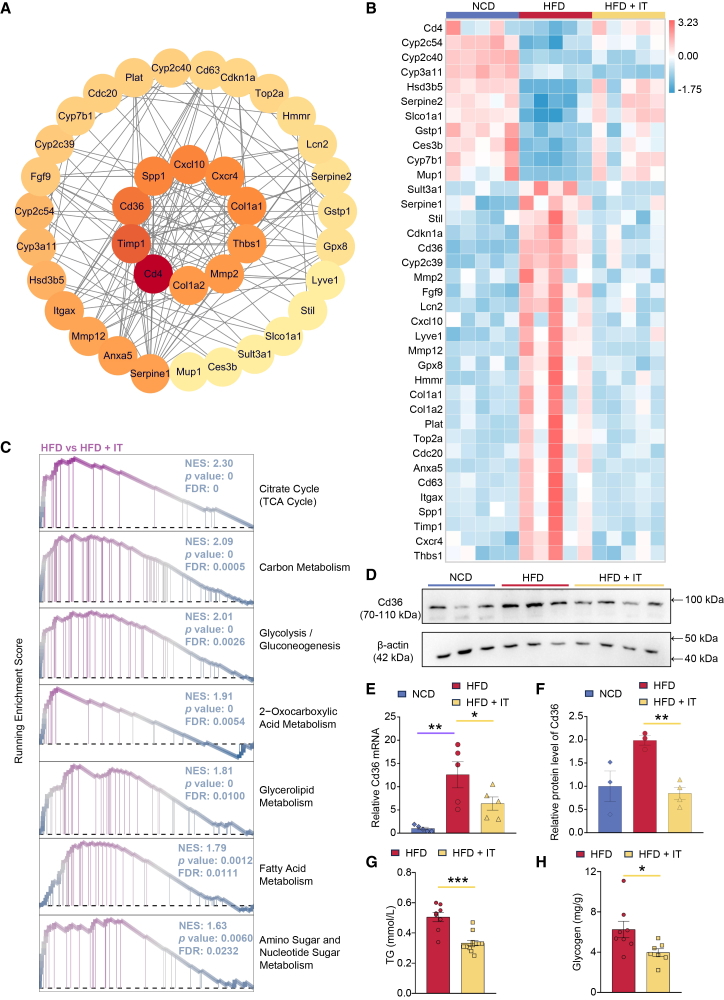
Identification of Cd36 as a central regulatory node in iturin’s action All data presented herein were generated from samples collected in the first experimental series/therapeutic model. (A) The core targets of iturin analyzed by Cytoscape. Node color represents degree centrality. (B) The heatmap depicting the fpkm of core targets of iturin (*n* = 5 per group). (C) KEGG pathways positively correlated with *Cd36* analyzed by GSEA. (D) Representative western blot images of Cd36 protein in liver tissues (*n* = 3–4 per group). The blots were probed with antibodies against Cd36 and β-actin. Cd36 and β-actin bands migrated at approximately 70–110 kDa and 42 kDa, respectively. (E) The relative mRNA level of *Cd36* tested by qRT-PCR (*n* = 5 per group). Data are presented as mean ± SEM. Statistical significance was determined by unpaired Student’s *t* test, comparing the HFD group with the NCD group, and the HFD + IT group with the HFD group. ∗*p* < 0.05 and ∗∗*p* < 0.01. (F) Quantitative analysis of Cd36 protein expression from (D) (*n* = 3–4 per group). Data are presented as mean ± SEM. Statistical significance was determined by unpaired Student’s *t* test, comparing the HFD group with the NCD group, and the HFD + IT group with the HFD group. ∗∗*p* < 0.01. (G) The levels of serum triglyceride (TG) (*n* = 8–12 per group). Data are presented as mean ± SEM. Statistical significance was determined by unpaired Student’s *t* test, comparing the HFD + IT group with the HFD group. ∗∗∗*p* < 0.001. (H) The levels of hepatic glycogen (*n* = 7–8 per group). Data are presented as mean ± SEM. Statistical significance was determined by unpaired Student’s *t* test, comparing the HFD + IT group with the HFD group. ∗*p* < 0.05.

A total of 274 DEGs were identified as common between the NCD group and the HFD group, as well as between the HFD group and the IT-supplemented group (Figure S6A). These genes were analyzed using Cytoscape software, initially yielding 183 nodes and 636 edges. Core targets were identified using Centiscape 2.2, resulting in the selection of 37 targets, which corresponded to 37 nodes and 164 edges (Figures S6B and S6C). Among these core targets, the top ten genes were *Cd4*, *Timp1*, *Cd36*, *Spp1*, *Cxcl10*, *Cxcr4*, *Col1a1*, *Thbs1*, *Mmp2*, and *Col1a2* (Figure 6A). Notably, *Cd4* expression was upregulated, whereas *Timp1*, *Cd36*, *Spp1*, *Cxcl10*, *Cxcr4*, *Col1a1*, *Thbs1*, *Mmp2*, and *Col1a2* were downregulated following IT administration (Figure 6B). According to data from the Liver Cell Atlas (https://www.livercellatlas.org) and the fpkm values from RNA-seq, among these ten targets, *Cd36* is highly expressed in hepatocytes, while the expression of other genes is relatively low (Figures S7A–S7K). Consequently, IT may influence glycolipid metabolism in the mouse liver primarily through *Cd36*. Gene set enrichment analysis (GSEA) revealed a positive correlation between *Cd36* expression and various metabolic pathways, including the citrate cycle, carbon metabolism, glycolysis/gluconeogenesis, 2-oxocarboxylic acid metabolism, glycerolipid metabolism, fatty acid metabolism, and amino sugar and nucleotide sugar metabolism (Figure 6C). Conversely, *Cd36* was negatively correlated with pathways such as steroid hormone biosynthesis, arachidonic acid metabolism, linoleic acid metabolism, retinol metabolism, tryptophan metabolism, primary bile acid biosynthesis, and peroxisome function (Figure S8). Consequently, pathways that were positively correlated with *Cd36* experienced downregulation, whereas those negatively correlated with *Cd36* were upregulated (Figures S9A and S9B). The downregulation of Cd36 following IT supplementation was confirmed at both the transcriptional and translational levels (Figures 6D–6F). RT-PCR analysis demonstrated a significant reduction in hepatic *Cd36* mRNA expression in IT-treated mice compared to HFD controls. Consistent with this finding, western blot analysis further revealed that the elevated hepatic Cd36 protein expression induced by HFD feeding was markedly attenuated by IT supplementation. Furthermore, the supplementation of IT led to a reduction in TG content and the accumulation of glycogen in the liver (Figures 6G and 6H). In aggregate, IT downregulated *Cd36* and orchestrated glycolipid metabolic homeostasis.

To elucidate the direct role of Cd36 in mediating the lipid-lowering effect of IT, an *in vitro* inhibition experiment was conducted using the Cd36-specific inhibitor sulfosuccinimidyl oleate (SSO) in HepG2 cells. The cells were allocated into five experimental groups: untreated control, PA/OA-induced steatosis model, PA/OA-induced model co-treated with IT, PA/OA-induced model co-treated with SSO, and PA/OA-induced model co-treated with both SSO and IT. Oil Red O staining revealed that PA/OA treatment induced prominent intracellular lipid deposition. This lipid accumulation was significantly attenuated by co-treatment with IT. SSO treatment alone also partially reduced lipid accumulation compared to the model group. Notably, the combination of SSO and IT did not yield an additive effect; the extent of lipid reduction was comparable to that achieved with SSO treatment alone (Figure S10). These findings suggest that IT ameliorates hepatic steatosis, at least in part, by modulating the Cd36 pathway.

### Iturin modulates the amino sugar and nucleotide sugar metabolism pathway

**Figure 7:**
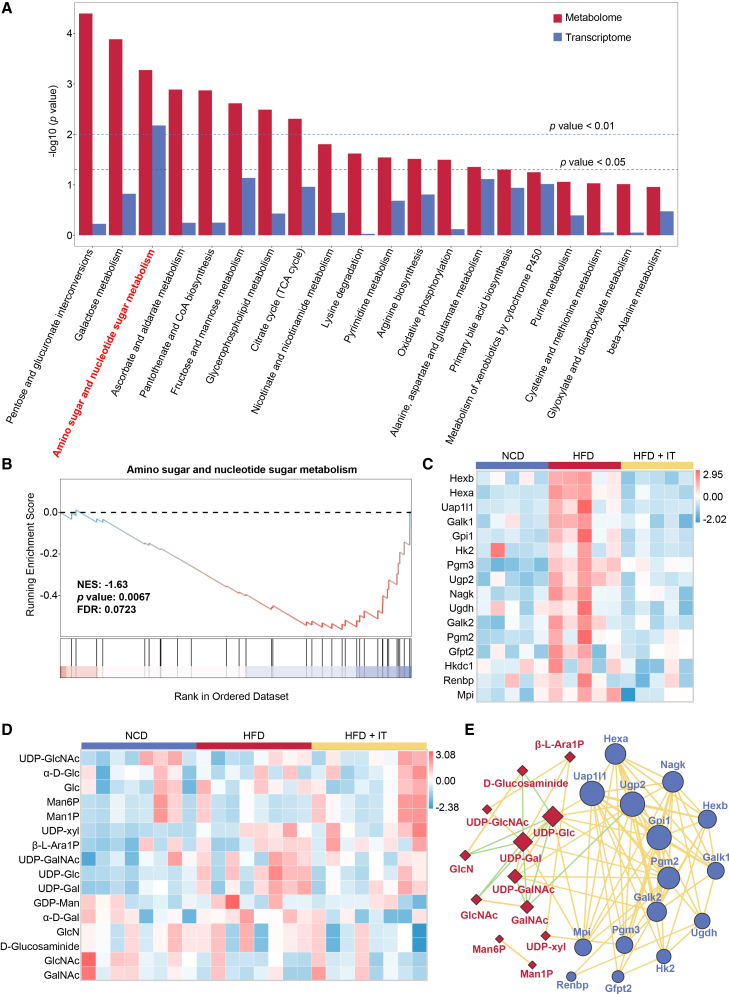
Integrated multi-omics analysis implicates the amino sugar and nucleotide sugar metabolism pathway All data presented herein were generated from samples collected in the first experimental series/therapeutic model. (A) Integrated metabolome-transcriptome mapping of KEGG pathways following itrurin supplementation. Bar chart displays the KEGG pathways enriched by integrating significantly altered genes (|log2 fold change| > 1, adjusted *p* value <0.05) and metabolites (|log_2_ fold change| > 1, adjusted *p* value <0.05). The bar height represents the -log_10_ (*p* value) of pathway enrichment. (B) The pathway of amino sugar and nucleotide sugar metabolism analyzed by GSEA. GSEA was performed on the pre-ranked gene list (by log_2_ fold change) using the KEGG gene set collection. (C) The heatmap depicting the fpkm of leading edge genes in amino sugar and nucleotide sugar metabolism pathway (*n* = 5 per group). (D) The heatmap depicts the metabolites involved in the amino sugar and nucleotide sugar metabolism pathway (*n* = 8 per group). (E) Correlation network between leading-edge genes (blue circles) and metabolites (red diamonds) involved in the amino sugar and nucleotide sugar metabolism pathway. Significant pairwise *Pearson’s* correlations are shown (|*r* | > 0.7, *p* < 0.05). Yellow lines indicate positive correlations (*r* > 0.7), and green lines indicate negative correlations (*r* < −0.7).

A comprehensive analysis of the metabolome and transcriptome demonstrated that IT supplementation influenced the amino sugar and nucleoside sugar metabolic pathways at both metabolic and transcriptional levels (Figure 7A). This finding was corroborated by GSEA, which identified core enrichment genes such as *Hexb*, *Hexa*, *Uap1l1*, *Galk1*, *Gpi1*, *Hk2*, *Pgm3*, *Ugp2*, *Nagk*, *Ugdh*, *Galk2*, *Pgm2*, *Gfpt2*, *Hkdc1*, *Renbp*, and *Mpi* (Figures 7B and 7C). Within this pathway, metabolites such as uridine 5′-diphospho-glucose (UDP-Glc) and galactose (α-D-Gal), which are involved in glycogen synthesis or energy metabolism, demonstrated a downward trend, whereas mannose-1-phosphate/mannose-6-phosphate (Man1P/Man6P), not typically associated with energy metabolism, demonstrated an increasing tendency (Figure 7D). Multi-component analysis revealed a positive correlation between UDP-Glc and genes *Uap1l1*, *Ugp2*, *Gpi1*, and *Pgm2* (Figure 7E). Furthermore, KEGG pathway analysis identified *Ugp2* as a crucial enzyme that catalyzes the conversion of glucose-1-phosphate and UTP into UDP-Glc (Figure S11).

Non-targeted metabolomic studies encounter significant technical challenges in differentiating structural and conformational isomers within carbohydrate superfamilies. This is particularly evident in the inadequate resolution required to distinguish epimers, such as glucose and galactose, and anomers with α/β configurations. These technical constraints can result in false-negative identifications and quantitative biases during carbohydrate screening. For instance, the *Hk2* gene, responsible for phosphorylating glucose to glucose-6-phosphate, was found to be significantly downregulated in RNA-seq analyses of mouse liver, a finding corroborated in HepG2 cells (Figure S12A). Additionally, the supplementation of IT led to the upregulation of *G6pd*, which channels glucose-6-phosphate toward the pentose phosphate pathway rather than glycogen synthesis (Figure S12B). This suggests that after IT supplementation, carbohydrates are more likely to be directed toward non-glycogen storage and non-energy supply pathways. However, sparse correlational evidence was identified in the metabolomic dataset (Figure S12C). Therefore, more precise experimental investigations are required to elucidate the effects of IT on carbohydrate regulation following supplementation.

Interestingly, the expression of most members of the lipocalin superfamily, particularly MUPs, was significantly increased in mice supplemented with IT (Figures S13A–S13D). Among these members, *Mup1*, a key member of the MUPs, demonstrated a marked decrease in obese mice, and its upregulation resulted in the suppression of gluconeogenic and lipogenic gene expression in the livers of mice exposed to a HFD.^18^ Additionally, treatment with recombinant MUP1 significantly improved hyperglycemia, glucose tolerance, and insulin sensitivity in mice.^19^ However, the precise mechanisms through which *Mup1* is involved in these processes remain unclear.

Consequently, IT modulated metabolism via the amino sugar and nucleoside sugar metabolism pathway, potentially directing carbon compounds toward non-glycogen or non-energy metabolic pathways. Moreover, IT may play a role in modulating glycolipid metabolism through the Mups family.

### Iturin induces modifications in the gastrointestinal microbiome of mice

To evaluate potential structural changes in the intestine within the gut-liver axis, histological examination of Swiss-rolled intestinal segments was performed via H&E staining. No overt alterations in intestinal architecture were observed among the NCD-fed, HFD-fed, and IT-supplemented HFD-fed groups, indicating that the pathological development of MASLD and the subsequent intervention were not associated with gross intestinal morphological injury (Figure S14).

**Figure 8:**
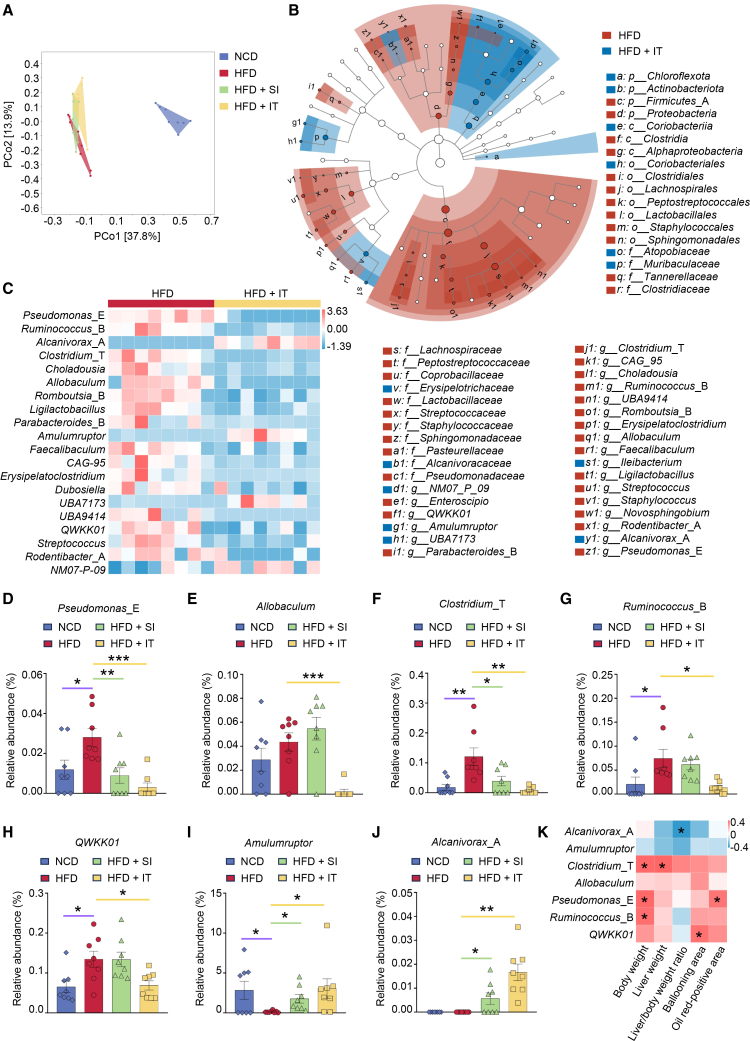
Iturin supplementation modulates the gut microbiota composition in HFD-fed mice All data presented herein were generated from samples collected in the first experimental series/therapeutic model. (A) Principal coordinates analysis (PCoA) of gut microbiota at the ASV/OTU level using the Bray-Curtis distance algorithm (*n* = 8 per group). (B) LefSe analysis of the gut microbiota in HFD-fed mice subjected to either saline or iturin treatment (LDA score >2). (C) The heatmap depicts the major distinctions in gut microbial composition among HFD-fed mice subjected to either saline or iturin treatment (*n* = 8 per group). (D–I) The relative abundance of *Pseudomonas*_E (D), *Allobaculum* (E), *Clostridium*_T (F), *Ruminococcus*_B (G), *QWKK01* (H), *Amulumruptor* (I), and *Alcanivorax*_A (J) (*n* = 8 per group). Data are presented as mean ± SEM. Statistical significance was determined by unpaired Student’s *t* test, comparing the HFD group with the NCD group, and the treatment group (HFD + SI, HFD + IT) with the HFD group. ∗*p* < 0.05, ∗∗*p* < 0.01, and ∗∗∗*p* < 0.001. (K) The *Spearman’s* correlation coefficient between gut microbial composition and the index of MASLD. ∗*p* < 0.05.

The composition of the gut microbiota is instrumental in the pathogenesis of MASLD, with discernible alterations observed in both human patients and murine models. In the current study, 16S rRNA sequencing was employed to scrutinize alterations in the gut microbiota of mice following supplement with IT, using samples from the first experiment (therapeutic intervention). HFD-fed mice exhibited reduced microbial diversity and abundance compared to NCD-fed controls (Figure S15). Dietary IT intervention modulated gut microbiota composition of mice, with IT-supplemented mice exhibiting 559 unique species (ASV/OTU) compared to HFD-fed mice administered with SA, among which 38 species were shared with NCD-fed mice (Figure 8A). Differential microbial taxa in mice supplemented with IT, as revealed by LefSe analysis, encompassed 4 phyla and 23 genera compared to HFD-fed mice administered with SA (Figure 8B). Genus-level analysis of gut microbiota composition delineated shifts in the relative abundance of *Pseudomonas*_E (Figures 8C and 8D), *Allobaculum* (Figures 8C–8E), *Clostridium*_T (Figures 8C–8F), *Ruminococcus*_B (Figures 8C–8G), *QWKK01* (Figures 8C–8H), which were elevated in HFD-fed mice but significantly reduced in IT-supplemented HFD-fed mice. Conversely, *Amulumruptor* exhibited decreased abundance in HFD-fed mice but increased levels in IT-supplemented HFD-fed mice (Figures 8D–8I), while *Alcanivorax*_A was uniquely detected in mice receiving IT, but not in SA-administered mice, either NCD-fed or HFD-fed mice (Figures 8C–8J). To investigate the association between microbiota and MASLD, Spearman’s correlation analysis was performed to assess their relationship with MASLD-related parameters. The results demonstrated a positive correlation between *Pseudomonas*_E and mouse body weight as well as lipid deposition, whereas *Clostridium*_T displayed a positive relationship with both liver weight and body weight. Additionally, *Ruminococcus*_B demonstrated a positive correlation with body weight. Furthermore, *QWKK01* exhibited a positive correlation with the ballooning index. Conversely, a negative correlation was identified between *Alcanivorax*_A and the liver weight/body weight ratio (Figure 8K).

Bile acids exhibit a significant relationship with the intestinal microbiota. Primary bile acids are synthesized in the liver, while secondary bile acids are produced through the action of the intestinal microbiota. RNA-seq data indicated that the primary bile acid synthesis pathway was influenced by the addition of IT (Figure S16A), leading to the upregulation of the key enzyme *Cyp7b1* involved in bile acid synthesis (Figure S16B). This finding was corroborated by RT-PCR results (Figure S16C). Metabolomic analyses further revealed that the supplementation of IT resulted in an increased concentration of bile acids, including taurocholic acid and tauroursodeoxycholic acid. The relationship between bile acids and the intestinal microbiota was assessed using Spearman correlation analysis. The genera *Pseudomonas*_E, *Allobaculum*, and *Ruminococcus*_B demonstrated a positive correlation with bile acid content, whereas *Alcanivorax*_A exhibited a negative correlation. *Clostridium*_T exhibited a negative correlation exclusively with taurocholic acid content, whereas *Amulumruptor* demonstrated a positive correlation solely with taurocholic acid content. No significant correlations were observed between either *Clostridium*_T or *Amulumruptor* and taurodeoxycholic acid content. Additionally, *QWKK01* was negatively correlated with tauroursodeoxycholic acid content but showed no correlation with taurocholic acid content (Figure S16D).

Consequently, IT has the potential to modulate the gut microbiota by increasing the prevalence of beneficial bacteria associated with MASLD and decreasing the abundance of harmful bacteria linked to the condition. The incorporation of IT may also influence bile acid metabolism through its impact on intestinal flora.

## Discussion

In this study, IT was observed to impart hepatoprotective properties and modulate glucose and lipid metabolism (Figures 1, 2, 3, 4, and 5). It demonstrated notable efficacy in ameliorating fatty liver conditions and inhibiting hepatic lipid accumulation. Furthermore, IT effectively mitigated HFD-induced body weight gain in mice. The integration of multi-omics analyses (transcriptomics, metabolomics, 16S rRNA sequencing) with targeted functional validation reveals that its therapeutic efficacy against MASLD involves a multi-target mechanism, with a key point of convergence being the downregulation of hepatic *Cd36*. From the RNA-seq results, 37 core targets of IT were identified, encompassing genes involved in lipid metabolism (e.g., *Cd36* and *Lcn2*), inflammation and immunity (e.g., *Cd4* and *Cxcl10*), fibrosis and extracellular matrix remodeling (e.g., *Col1a1*, *Timp1*, and *Spp1*), oxidative stress (e.g., *Gstp1*), and bile acid metabolism (e.g., *Cyp7b1*) (Figures 6A and S6). This network underscores the pleiotropic nature of IT’s action against the multifaceted pathophysiology of MASLD.

Among the core targets, *Cd4* displays the highest connectivity. As a key immune molecule predominantly expressed in hepatic immune cells,^20^ its expression is reduced in the context of MASLD.^21^ Given the association between MASLD and increased liver cancer risk,^22^ and the recognized role of CD4^+^ T cells in inhibiting tumor progression,^23^ the restoration of *Cd4* expression by IT (Figures 6A and 6B) suggests a potential immunomodulatory benefit.

*Timp1*, the second most interconnected molecule, is a critical marker of liver injury and fibrosis that is minimally expressed in the healthy liver.^24^ It is upregulated during hepatic stellate cell activation and ECM remodeling.^25^ IT administration significantly lowered hepatic *Timp1* expression (Figures 6A and 6B), indicating attenuated liver injury. Despite the higher node connectivity of *Cd4* and *Timp1*, both molecules exhibit relatively low expression in the liver, as evidenced by data from the Liver Cell Atlas public database and RNA-seq analyses. The observed modulation of Cd4 and Timp1 by IT likely reflects secondary improvements consequential to the amelioration of core metabolic disturbances and hepatic steatosis, rather than representing primary direct targets. This interpretation aligns with their low basal hepatic expression and the primary metabolic focus of this intervention.

Among these targets, *Cd36* emerged as a central mechanistic hub. *Cd36* is a membrane receptor and transporter protein that plays a crucial role in facilitating the transmembrane transport of long-chain fatty acids (LCFAs), regulating lipid synthesis and oxidation, mediating inflammatory signal transduction, interfering with insulin signaling, and promoting hepatic glycogenesis.^26^ The expression levels of *Cd36* in the liver have been positively correlated with TG levels, and patients exhibiting high *Cd36* expression are more likely to progress from simple fatty liver to hepatitis or cirrhosis.^27^ In mice, hepatic *Cd36* levels were significantly elevated when fed a HFD compared to those fed a normal diet. Notably, the addition of IT to the diet resulted in a significant decrease in *Cd36* levels (Figures 6A, 6B, and 6D). Consequently, IT may further modulate glycolipid metabolism through its effects on *Cd36*. Critically, IT supplementation significantly downregulated hepatic *Cd36* at both mRNA and protein levels in HFD-fed mice (Figures 6D–6F). Most importantly, the functional relevance of *Cd36* modulation was established *in vitro*: In a PA/OA-induced HepG2 steatosis model, the lipid-lowering effect of IT was non-additive with that of the specific *Cd36* inhibitor SSO, strongly suggesting that IT alleviates hepatic lipid accumulation primarily through the *Cd36* pathway (Figure S10). This series of evidence solidifies *Cd36* downregulation as the pivotal event mediating IT’s primary lipid-lowering effect.

GSEA was employed to examine the KEGG pathways associated with *Cd36*. The findings corroborated the hypothesis, demonstrating a significant positive correlation between *Cd36* and several metabolic pathways, including the TCA cycle, carbon metabolism, glycolysis/gluconeogenesis, fatty acid metabolism, and amino sugar and nucleotide sugar metabolism (Figure 6C). Furthermore, an integrated metabolomics and transcriptomics analysis revealed that the addition of IT markedly influenced the amino sugar and nucleotide sugar metabolism pathway (Figure 7A). This pathway represents a crucial branch of glucose metabolism, interfacing with glycolysis, the pentose phosphate pathway, and lipid metabolism, thereby serving as a nexus between energy production and metabolic processes. Analysis of various metabolites indicated a reduction in the levels of UDP-Glc and α-D-Gal, which are involved in energy supply and glycogen synthesis, alongside an increase in carbohydrates not participating in energy metabolism following IT supplementation (Figure 7D). Consequently, IT may further impact the amino sugar and nucleotide sugar metabolism pathway by down-regulating *Cd36*, leading to decreased glycogen synthesis and energy supply, ultimately contributing to reduced hepatic lipid accumulation.

In addition to influencing carbohydrate and lipid metabolism, IT altered levels of key metabolites implicated in MASLD pathophysiology, including bile acids, glutathione, NAD+, and vitamin/cofactor precursors (Figures 3C–3E). IT upregulated *Cyp7b1* (Figures S16A–S16C), a key enzyme in the alternative bile acid synthesis pathway, and increased hepatic levels of muricholic acid, taurocholic acid, and tauroursodeoxycholic acid (Figure 3E). Bile acids are crucial regulators of glucose and lipid metabolism.^28^ Tauroursodeoxycholic acid is linked to ER stress and MASLD.^29^ While the relationship between taurocholic acid and MASLD remains unreported, our research suggests that taurocholic acid may also play a role in the advancement of MASLD and could represent a promising therapeutic target for the disease.

IT elevated hepatic glutathione, a major antioxidant,^30^ likely by upregulating *G6pd* (Figure S12B) to shunt glucose-6-phosphate into the pentose phosphate pathway for NADPH generation, supporting glutathione regeneration.^31^ Levels of NAD+, a central redox cofactor and promising therapeutic target in MASLD,^32^ and its derivative cyclic ADP-ribose were increased by IT (Figure 3E). IT also raised levels of gulono-1,4-lactone (a vitamin C precursor) and pantothenic acid (a coenzyme A precursor) (Figure 3E). Vitamin C acts as an antioxidant and modulates lipid metabolism,^33^ while coenzyme A is essential for the TCA cycle and fatty acid oxidation.

IT suppressed harmful gut bacteria linked to MASLD. Specifically, it reduced levels of *Pseudomonas*_E (Figures 8C and 8D), a Gram-negative opportunistic pathogen,^34^ and *QWKK01* (Figures 8C–8H), a member of *Eggerthellaceae* that is elevated in MASLD and may influence short-chain fatty acid (SCFA) production.^35^ The presence of detrimental bacteria such as *Pseudomonas*_E may contribute to MASLD onset, as the disease increases susceptibility to infections.^36^ IT also reduced the HFD-elevated abundance of *Allobaculum* (Figures 8C–8E), a butyrate producer.^37^

IT modulated beneficial bacteria associated with intestinal health. These include *Romboutsia* and *Ruminococcus*, which help safeguard intestinal barrier function and immune equilibrium,^38^ and *Clostridium*, a key producer of bile salt hydrolase essential for bile acid metabolism.^39^ An increase in these microbes is suggested to be protective in early MASLD.^40^ Notably, while their abundance increased after HFD feeding, it decreased following IT treatment alongside MASLD amelioration (Figures 8B and 8C, 8F, and 8G).

IT also increased the abundance of *Amulumruptor* (Figures 8C–8I), which is implicated in carbohydrate metabolism,^41^ and *Alcanivorax* (Figures 8C–8J), which contributes to carbon cycling.^42^^,^^43^ Nevertheless, there remains a limited comprehension of the roles played by *Amulumruptor* and *Alcanivorax* in the pathogenesis of MASLD and the mechanisms governing *Alcanivorax* production. Beyond direct metabolic regulation, IT exerted a multifaceted modulatory effect on the gut-liver axis. IT induced a favorable restructuring of the gut microbiota. Notably, these substantial and specific microbial shifts were observed under the experimental condition where overt intestinal structural damage was absent across all groups, including the HFD-fed mice (Figure S14). Therefore, the beneficial modulation by IT represents a targeted biological effect on the gut microbiota composition and function, rather than a secondary consequence of altered intestinal morphology.

In conclusion, this study positions IT as a multi-target dietary bioactive agent effective against diet-induced MASLD. By integrating multi-omics and functional validation, we delineate a mechanism centered on the downregulation of hepatic *Cd36*, which orchestrates a broad metabolic reprogramming and is synergized by beneficial remodeling of the gut microbiota. This pleiotropic action profile, which simultaneously addresses lipid uptake, oxidative stress, and microbial ecology, aligns well with the complex pathogenesis of MASLD. Our work not only highlights the therapeutic promise of IT but also provides a systems-level framework for understanding how microbial metabolites can intercept metabolic disease.

### Limitations of the study

While this study provides comprehensive evidence for the anti-MASLD effects of IT, several limitations should be acknowledged. First, although our *in vitro* data using a Cd36 inhibitor support the involvement of this pathway, definitive *in vivo* causal evidence through hepatocyte-specific Cd36 knockout or overexpression models is required to unequivocally establish Cd36 as the indispensable mediator of IT’s effects. Second, the precise molecular mechanism by which IT downregulates Cd36 expression remains unexplored; future studies should investigate whether IT directly interacts with signaling upstream of Cd36 (e.g., PPARγ and LXR) or modulates its stability. Moreover, while our data demonstrate improved systemic lipid profiles (e.g., LDL-C/HDL-C), further investigations into more dynamic metabolic parameters, including circulating FFA flux and tissue insulin sensitivity, are warranted to fully elucidate the effects of IT on lipid mobilization and utilization. Third, the current PK analysis is preliminary; a full-time-course PK study with detailed tissue distribution, especially in the liver and intestine, is needed to fully understand its bioavailability and target organ exposure. Specifically, the selection of the IT dose (14 mg/kg) in this exploratory study was primarily based on safety considerations and literature precedent for bioactive peptides, rather than on established dose-response or PK data specific to MASLD. While our preliminary single-time-point distribution study confirms IT’s oral bioavailability and presence in key tissues such as the liver, it does not provide information on critical PK parameters (e.g., C_max_, T_max_, half-life, and AUC) or define the optimal therapeutic window. The lack of comprehensive PK profiling and dose-ranging experiments represents a significant translational gap, as it precludes a clear understanding of the relationship between administered dose, systemic and hepatic exposure, and the observed therapeutic effects. Future studies incorporating systematic dose-response assessments and full-time-course PK analyses are essential to establish the compound’s pharmacological profile and guide its potential development. Fourth, it should be acknowledged that the pharmacological characterization of IT in this study is constrained by the unavailability of a certified commercial standard. Without an absolute reference standard, we could not perform absolute quantification or establish a validated calibration curve for IT. Consequently, the compound’s purity was estimated by relative peak area normalization (as detailed in the method details), and batch-to-batch consistency was ensured by matching chromatographic and mass spectrometric profiles to a reference batch (Figure S2). While this approach is widely accepted for purified microbial metabolites and provides a robust internal consistency control, it does not allow for the determination of exact molar concentrations in biological samples or the establishment of internationally harmonized quality specifications. Future studies incorporating a certified IT standard are essential to perform absolute quantification, establish rigorous PK-pharmacodynamic relationships, and define standardized quality control protocols for potential therapeutic development. Fifth, while 16S rRNA sequencing revealed compositional changes in the gut microbiota, metagenomic or metatranscriptomic analyses would be necessary to delineate the functional consequences of these shifts and identify specific bacterial genes or metabolites (e.g., secondary bile acids and SCFAs) that mediate the systemic benefits. Sixth, the assessment of intestinal integrity was limited to histological examination, which revealed no overt structural damage. Functional assessments of the gut barrier, such as permeability to macromolecules (e.g., via FITC-dextran assay) or the expression of key tight-junction proteins, were not performed. Given the established link between gut permeability, metabolic endotoxemia, and MASLD progression, future studies incorporating these functional assessments are needed to determine whether the IT-induced microbial remodeling also translates into improved gut barrier integrity. Finally, the long-term safety and efficacy of IT supplementation, as well as its effects on advanced stages of MASLD such as steatohepatitis and fibrosis, warrant investigation in longer-duration preclinical models.

## Resource availability

### Lead contact

Requests for further information and resources should be directed to and will be fulfilled by the lead contact, Hongwei Liu (lhwei1987@126.com).

### Materials availability

This study did not generate any unique reagents.

### Data and code availability

• Transcriptomics and 16S rRNA gene sequencing data have been deposited at the NCBI BioProject database under accession numbers PRJNA1131084, PRJNA1131146, and PRJNA1131181, and are publicly available as of the date of publication.
• This paper does not report original code.
• Any additional information required to reanalyze the data reported in this paper is available from the lead contact upon request.

## Acknowledgments

This work was supported by the 10.13039/501100012166Province Key R&D Program of Hebei (no. 23372401D), 10.13039/501100012245Science and Technology Planning Project of Hebei Academy of Sciences (25308), and High-Level Talents Training and Funding Projects of 10.13039/501100014898Hebei Academy of Sciences (2024G11).

## Author contributions

L.Y.Z.: writing – original draft, writing – review and editing, investigation, and data curation. Q.L.: investigation and data curation. J.N.H.: investigation and data curation. Y.J.L.: investigation. Y.Z.: investigation. Y.X.F.: investigation. W.Y.Z.: investigation. L.P.Z.: investigation. Y.F.L.: investigation and project administration. T.L.L.: investigation and project administration. H.W.L.: writing – review and editing, investigation, data curation, funding acquisition, and project administration.

## Declaration of interests

H.W.L., L.Y.Z., Y.F.L., and L.P.Z. are inventors on patents describing the utilization of iturin family peptides in pharmaceutical formulations for the prevention and/or treatment of non-alcoholic fatty liver disease (Chinese Patent ZL202411089240.3; Dutch Patent NL2039994A).

## STAR★Methods

### Key resources table

**Table:** 

REAGENT or RESOURCE	SOURCE	IDENTIFIER
**Antibodies**		

Anti-Cd36 antibody (rabbit monoclonal)	Abcam	ab252923; RRID: AB_3678970
Anti-β-actin antibody (mouse monoclonal)	Abcam	ab8227; RRID: AB_2305186
HRP-conjugated goat anti-rabbit IgG	Abcam	ab6721; RRID: AB_955447

**Bacterial and virus strains**		

*Bacillus velezensis*	This study	N/A

**Chemicals, peptides, and recombinant proteins**		

Hydrochloric acid (HCl)	Tianjin Bodi Chemical Co., Ltd.	N/A
Methanol	Thermo Fisher	A452-4
Palmitic acid (PA)	MCE	HY-N0830
Oleic acid (OA)	MCE	HY-N1446
Dimethyl sulfoxide (DMSO)	Sigma-Aldrich	D2650
Sulfo-N-succinimidyl oleate (SSO)	AbMole	M13575
Acetonitrile	Fisher Optima	A955-4
Formic acid	Fisher Optima	A117-50
Paraformaldehyde (PFA)	Sigma-Aldrich	P6148
Ethanol	Sigma-Aldrich	459836
Xylene	Fisher Scientific	X3P-1GAL
Paraffin	Sigma-Aldrich	P3681
Hematoxylin solution	Solarbio	G1120
Eosin	Solarbio	G1120
Sucrose	Servicebio	G3010
OCT compound	Sakura	4583
Oil Red O solution	Servicebio	G1015
Dulbecco‘s Modified Eagle Medium (DMEM)	Gibco	11965092
Fetal bovine serum (FBS)	Gibco	10099141
0.9% saline (SA)	Shijiazhuang No.4 Pharmaceutical Co., Ltd.	2304231503
Silybin (SI)	Xi’an Green Biotech Co., Ltd.	GL20230511
Iturin (IT)	Isolated from *B. velezensis* in this study	N/A

**Critical commercial assays**		

Liver/Muscle Glycogen Assay Kit	Nanjing Jiancheng Bioengineering Institute	A043-1-1
Trizol Reagent	Invitrogen Life Technologies	15596026

**Deposited data**		

Metabolomics data	This study	Data available upon request
Transcriptomics data	This study	PRJNA1131084, PRJNA1131146
16S rRNA sequencing data	This study	PRJNA1131181

**Experimental models: Cell lines**		

HepG2 human hepatoma cells	ATCC	HB-8065

**Experimental models: Organisms/strains**		

C57BL/6J male mice	Gempharmatech Co., Ltd.	SCXK(Jing)2023-0008

**Oligonucleotides**		

qPCR primers for Cd36, see Table S1	This study	Custom synthesis
qPCR primers for Mup1, see Table S1	This study	Custom synthesis
qPCR primers for Mup16, see Table S1	This study	Custom synthesis
qPCR primers for Mup14, see Table S1	This study	Custom synthesis
qPCR primers for Plin4, see Table S1	This study	Custom synthesis
qPCR primers for Lcn2, see Table S1	This study	Custom synthesis
qPCR primers for Cyp7b1, see Table S1	This study	Custom synthesis
qPCR primers for Gstm3, see Table S1	This study	Custom synthesis
qPCR primers for Gpx3, see Table S1	This study	Custom synthesis
qPCR primers for Foxq1, see Table S1	This study	Custom synthesis
qPCR primers for Pnpla3, see Table S1	This study	Custom synthesis
qPCR primers for Gpat3, see Table S1	This study	Custom synthesis
qPCR primers for Ces3b, see Table S1	This study	Custom synthesis
qPCR primers for Aldh1b1, see Table S1	This study	Custom synthesis
qPCR primers for Hsd3b5, see Table S1	This study	Custom synthesis
qPCR primers for Cyp4a12b, see Table S1	This study	Custom synthesis
qPCR primers for Apoa4, see Table S1	This study	Custom synthesis
qPCR primers for Actinb, see Table S1	This study	Custom synthesis

**Software and algorithms**		

XCMSPlus	Sciex	v3.6.3
MetDNA 2.0	N/A	https://metdna.zhulab.cn
MetaboAnalyst	McGill University	v5.0
GSEA	Broad Institute	v4.4.0
Cytoscape	Cytoscape Consortium	v3.9.1
ImageJ	National Institutes of Health	v1.53
QIIME2	N/A	v2023.2
GraphPad Prism	GraphPad Software	v9.0
GenesCloud tools	N/A	https://www.genescloud.cn
Wei Sheng Xin platform	N/A	https://www.bioinformatics.com.cn
DESeq2	Bioconductor	v1.40.2
RStudio	Posit	v2023.12.1

**Other**		

Normal-Caloric Diet (NCD)	Jiangsu Xietong Pharmaceutical Bio-engineering Co., Ltd.	N/A
High-Fat Diet (HFD)	Jiangsu Xietong Pharmaceutical Bio-engineering Co., Ltd.	N/A
ExionLC-AD HPLC system	AB SCIEX	N/A
AB SCIEX 5600+ TripleTOF mass spectrometer	AB SCIEX	N/A
Yuwell 680CR glucometer	Yuwell	N/A
Leica RM2016 pathology slicer	Leica Microsystems	N/A
Thermo CRYOSTAR NX50 freezing microtome	Thermo Fisher Scientific	N/A

### Experimental model and study participant details

#### Animals

The C57BL/6J male mice (aged 6-8 weeks) used in the study were purchased from Gempharmatech Co., Ltd (license No. SCXK[Jing]2023-0008). All mice utilized in this study adhered to the standards for animal welfare. The mice were housed in separate ventilated cages, with 4 mice per cage, provided with abundant food and water, and maintained at a temperature of 24.5 ± 0.5°C under a 12/12-hour light/dark cycle.

All experimental procedures were approved by the Institutional Ethics Review Board of the Institute of Biology, Hebei Academy of Sciences (Approval No. SWS00303; Date of approval: 10 March 2023) and conducted in accordance with the committee’s guidelines and relevant regulations. The authors confirm that all procedures involving animals were carried out in compliance with the ethical standards for animal experimentation and in accordance with the ARRIVE guidelines.

Only male mice were used in the study to control for the confounding effects of sexual dimorphism in metabolism. Consequently, potential sex-dependent effects could not be evaluated, which is a recognized limitation and an important avenue for future investigation.

#### Cell lines

HepG2 cells (ATCC, HB-8065) were cultured in Dulbecco’s Modified Eagle Medium (DMEM, Gibco, 11965092) supplemented with 10% fetal bovine serum (FBS) (Gibio, 10099141) at 37 °C in a 5% CO_2_ environment. The cell line was authenticated by short tandem repeat profiling and tested negative for mycoplasma contamination.

#### Bacterial strain

The *B. velezensis* BA-26 used for iturin production was isolated from the hillside of Zhangshiyan by our laboratory. It was identified as *B. velezensis* based on morphological, physiological, biochemical characterization, and 16S rDNA sequence analysis. Its genotype is wild-type. The strain is maintained in long-term storage at -80°C in Nutrient Broth (NB) medium containing 25% glycerol. For experiments, the strain was activated and cultured in NB medium at 32 °C with continuous shaking at 180 rpm for 48 hours.

#### Ethics statement

All experimental procedures were approved by the Institutional Ethics Review Board of the Institute of Biology, Hebei Academy of Sciences (Approval No. SWS00303; Date of approval: 10 March 2023).

### Method details

#### Iturin isolation and purification

Iturin was isolated from *B*. *velezensis* through a series of steps.^44^^,^^45^ The strain was cultured in Nutrient broth at 32 °C for 48 hours and the resulting supernatant was obtained by centrifugation (8000 g, 10 minutes), acidified to pH 2.0 with HCl (Tianjin Bodi Chemical Co., Ltd.), and left at 4 °C overnight. Subsequently, methanol (Thermo Fisher, A452-4) was utilized to extract the crude product, which was then subjected to purification via HPLC (ExionLC-AD) to yield iturin. The structure of iturin was confirmed using LC-MS analysis (AB SCIEX 5600+ TripleTOF). The chromatographic purity of the primary iturin peak was estimated to be >95% (Table S1), as determined by relative peak area normalization from analytical LC-MS, indicating homogeneity under the specified analytical conditions. Given the unavailability of a commercial certified standard for iturin, the purity was estimated using the relative peak area method, a widely accepted approach for the quantification of purified microbial metabolites when an absolute standard is not obtainable. To ensure batch-to-batch consistency, all iturin used in this study was derived from a single master stock of *B. velezensis*; identical fermentation and multi-step purification protocols were strictly followed for every batch. Furthermore, each final preparation batch was characterized by analytical LC-MS. A batch was used for subsequent experiments only when its chromatographic profiles and mass spectra matched those of the reference batch (Figure S2) and when the estimated purity of the primary iturin peak exceeded 95%, as calculated by relative peak area normalization.

#### Animal experiments

In the first experimental model, C57BL/6J male mice aged 6-8 weeks were fed either NCD as the control or HFD (Jiangsu Xietong Pharmaceutical Bio-engineering Co., Ltd.) for 14 weeks to induce MASLD. Subsequently, the mice were randomly divided into 4 groups, each consisting of a minimum of 8 mice. These four groups were administered orally with 0.9% saline (SA) (Shijiazhuang No.4 Pharmaceutical Co., Ltd., 2304231503), 57 mg/kg silybin (SI, diluted in 0.9% saline) (Xi’an Green Biotech Co., Ltd., GL20230511) or 14 mg/kg iturin (IT, diluted in 0.9% saline) daily for 4 weeks. Samples from this first experimental model were used for all subsequent metabolomics, transcriptomics, and microbiome analyses presented in this study.

In the second experimental model, C57BL/6J mice aged 6-8 weeks were randomly allocated into 4 groups, each comprising a minimum of 6 mice. The mice in these groups were fed NCD and provided with regular water or 0.1 g/L iturin solution for 8 weeks. Meanwhile, mice in the other two groups were fed HFD and given either regular water or 0.1 g/L iturin solution for an 8-week period.

#### Assessment of serum biochemical parameters and hepatic glycogen levels

Serum samples were obtained at the time of mice sacrifice. The quantification of serum triacylglycerol (TG), alanine aminotransferase (ALT), aspartate aminotransferase (AST), high-density lipoprotein cholesterol (HDL-C), and low-density lipoprotein cholesterol (LDL-C) levels was conducted through blood biochemical analysis by Shanghai Weice Biotechnology Co., Ltd. The liver tissues were gathered, and the hepatic glycogen content was quantified using the Liver/Muscle Glycogen Assay Kit (A043-1-1) provided by Nanjing Jiancheng Bioengineering Institute.

#### Oral glucose tolerance test

Mice were fasted (with free access to water) for approximately 16 hours. Following the fasting period, each mouse received an oral gavage of glucose at a dose of 2 g per kg body weight, administered as a 20% (w/v) aqueous solution. Blood samples were collected from the tail vein at 0 (baseline), 15, 30, 60, and 120 minutes post-glucose administration. Blood glucose levels were immediately measured using a commercial glucometer (Yuwell, 680CR, China).

#### HepG2 cell culture, treatment, and steatosis model induction

The HepG2 cells were treated with either 50 ng/mL IT, 200 ng/mL IT, or DMSO (Sigma-Aldrich, D2650) as a control. After 24 hours of culture, the cells were collected for subsequent studies.

A cellular steatosis model was established in HepG2 cells by treatment with a mixture containing 0.25 mM palmitic acid (PA; MCE, HY-N0830) and 0.5 mM oleic acid (OA; MCE, HY-N1446) for 24 hours. The cells were assigned to the following five groups: a control group maintained in normal medium; a model group treated with the PA/OA mixture alone; an iturin treatment group co-treated with PA/OA and IT (200 ng/mL); an SSO treatment group co-treated with PA/OA and the Cd36 inhibitor sulfo-N-succinimidyl oleate (SSO; AbMole, M13575; 30 μM); and a combined SSO and IT treatment group which received PA/OA, SSO, and IT at the specified concentrations. Following treatment, intracellular lipid accumulation was assessed by Oil Red O staining.

#### Metabolomic profiling and analyses

Liver samples or cell lysates were treated with an extraction solution composed of methanol, acetonitrile (Fisher Optima, A955-4), and water in a ratio of 4:4:1 followed by grinding. The resulting mixture was incubated at -20°C for 2 hours and then centrifuged to collect the supernatant. LC-MS/MS analyses were conducted using a UHPLC system (ExionLC AD, AB SCIEX, USA) equipped with an ACQUITY UPLC BEH Amide column (2.1 mm × 100 mm, 1.7 μm, Waters) coupled to a quadrupole time-of-flight mass spectrometer (TripleTOF 5600+, AB SCIEX, USA). The column temperature was maintained at 30 °C. Mobile phase A was acetonitrile containing 0.1% (volume ratio) formic acid (Fisher Optima, A117-50), and B was HPLC-grade water containing 0.1% (volume ratio) formic acid for positive (ESI+) modes. The flow rate was 0.3 mL/min and the gradient was set as follows: 0-1 minute: 5% B, 1-16 minutes: 5% B to 100% B, 16-18 minutes: 100% B, 18-18.1 minutes: 100% B to 5% B, and 18.1-23 minutes: 5% B. The injection volume was 2 μL.

The data acquisition was operated using the information-dependent acquisition (IDA) mode. The source parameters were set as follows: ion source gas 1 (GAS1), 35 psi; curtain gas (CUR), 30 psi; temperature (TEM), 550°C; declustering potential (DP), 80V; clion energy, 35V; and ion spray voltage floating (ISVF), +5500 V for positive mode. The TOF MS scan parameters were set as follows: mass range, 400-1600 Da; accumulation time, 0.15 s/spectra. The product ion scan parameters were set as follows: mass range, 100-1650 Da; accumulation time, 0.03 s/spectra. Subsequently, the acquired raw data underwent processing using XCMSPlus v3.6.3 for peak detection, extraction, alignment, integration, and annotation. MetDNA 2.0 and MetaboAnalyst 5.0 tools were utilized for metabolite annotation and data analysis.

#### Transcriptomics and RT-PCR

Total RNA was extracted utilizing the Trizol Reagent (Invitrogen Life Technologies, 15596026), followed by transcriptomics and RT-PCR analyses carried out by Shanghai Personal Biotechnology Co., Ltd. The data was analysed by GSEA 4.4.0 and Cytoscape 3.9.1.

#### Western blot analysis

Liver proteins were extracted using RIPA lysis buffer, and concentrations were determined by BCA assay. Equal amounts of protein (20 μg) were separated by 10% SDS-PAGE, transferred to PVDF membranes, and blocked with 5% non-fat milk. Membranes were incubated overnight at 4 °C with primary antibodies against Cd36 (1:1000, ab252923, Abcam) and β-actin (1:5000, ab8227, Abcam). After washing, membranes were incubated with an HRP-conjugated secondary antibody (1:5000, ab6721, Abcam) and visualized using ECL. Band intensity was quantified with ImageJ software.

#### Hematoxylin and eosin staining

Liver samples were harvested and rinsed with phosphate-buffered saline (PBS, pH 7.4) (Sigma-Aldrich, P5493). Colon tissues were longitudinally opened, rinsed with PBS, and then rolled from the distal to the proximal end into a “Swiss roll” configuration, which was secured before fixation. All tissues were fixed in 4% paraformaldehyde (PFA, diluted in PBS, pH 7.4) (Sigma-Aldrich, P6148) at room temperature (25 °C ± 1 °C) for 24 hours. Subsequently, the samples underwent dehydration with ethanol (Sigma-Aldrich, 459836) gradients, clearing with xylene (Fisher Scientific, X3P-1GAL), and embedding in paraffin (Sigma-Aldrich, P3681). The tissues were sectioned into 5 μm slices using a pathology slicer (Shanghai Leica Instrument Co., Ltd, RM2016) and stained with Hematoxylin solution (Solarbio, G1120) for 3-5 minutes and Eosin (Solarbio, G1120) for 15 seconds.

#### Oil red O staining

Liver samples were obtained and washed with PBS, followed by fixation in 4% PFA at 4 °C overnight. The livers underwent dehydration with graded sucrose (Servicebio, G3010), and were then embedded in a mixture of 30% sucrose solution and OCT (1:1) (Sakura, 4583) under low-temperature conditions using liquid nitrogen. The tissues were sectioned into 5 μm slices using a freezing microtome (Thermo, CRYOSTAR NX50) and stained with oil red O solution (Servicebio, G1015) for 8-10 minutes in the absence of light.

For HepG2 cells, after the aforementioned treatments, the cells were washed, fixed with 4% PFA, and directly stained with the filtered Oil Red O working solution. After staining, the cells were washed and immediately imaged under an inverted fluorescence microscope to capture the red lipid droplets.

#### 16s rRNA gene sequencing

Mouse fecal samples were obtained and subjected to 16s rRNA gene sequencing by Shanghai Personal Biotechnology Co., Ltd. Subsequently, the generated data was analyzed using QIIME2 software.

#### Pharmacokinetic analysis of iturin in mice

Male C57BL/6J mice were administered a single oral gavage of IT at a dose of 14 mg/kg or an equal volume of the vehicle alone as control. Two hours after administration, the mice were euthanized, and target tissues were collected. The freshly collected tissues were promptly homogenized in pre-cooled methanol. Following centrifugation, the supernatant was collected and directly subjected to analysis by LC-MS/MS.

### Quantification and statistical analysis

Statistical analyses were performed using GraphPad Prism, GenesCloud tools (https://www.genescloud.cn), and the Wei Sheng Xin platform (https://www.bioinformatics.com.cn/). Quantitative data are expressed as mean ± standard error of the mean (s.e.m.), represented by error bars in all figures. Continuous variables between two groups were compared using unpaired two-tailed Student’s *t-tests* for non-parametric data; multi-group comparisons employed *one-way ANOVA* with Bonferroni post hoc correction.

For non-targeted metabolomics, differential metabolite screening was performed using the Mann-Whitney U test, with significance defined by a fold change threshold of |log_2_ fold change| > 1 and a *p*-value threshold of < 0.05. The resulting *p*-values were further adjusted for multiple testing using the Benjamini-Hochberg procedure to control the false discovery rate (FDR). Metabolites meeting the criteria of |log_2_ fold change| > 1 and FDR-adjusted *p* value < 0.05 were identified as significantly differentially abundant.

RNA-seq raw read counts were analyzed for differential expression using the DESeq2 package in R, which employs a generalized linear model based on the negative binomial distribution. Significantly differentially expressed genes (DEGs) were defined as those with an absolute |log_2_ fold change| > 1 and an FDR-adjusted *p* value < 0.05, as calculated by DESeq2’s built-in statistical model and multiple testing correction.

Statistical significance thresholds were predefined as *p* < 0.05 (∗), *p* < 0.01 (∗∗), and *p* < 0.001 (∗∗∗), with exact *p*-values reported for all analyses.
