# Supplementary material for: Iturin derived from Bacillus modulates lipid and glucose metabolism while mitigating the progression of MASLD
Source: iScience. 2026 Mar 25;29(4):115470. doi: 10.1016/j.isci.2026.115470 (PMC13081174; doi:10.1016/j.isci.2026.115470)
Supplement: Document S1. Figures S1–S16 and Tables S1 and S2 [file mmc1.pdf]

iScience, Volume *29*

Supplemental information

Iturin derived from *Bacillus* modulates lipid and glucose metabolism while mitigating the progression of MASLD

Lingyun Zhao (赵凌云), Qing Liu (刘青), Jianuo He (贺佳诺), Yujian Li (李宇健), Yang Zhang (张阳), Yingxue Feng (冯映雪), Wenya Zhao (赵雯雅), Liping Zhang (张丽萍), Yongfeng Liu (刘永锋), Tongliang Li (黎彤亮), and Hongwei Liu (刘洪伟)

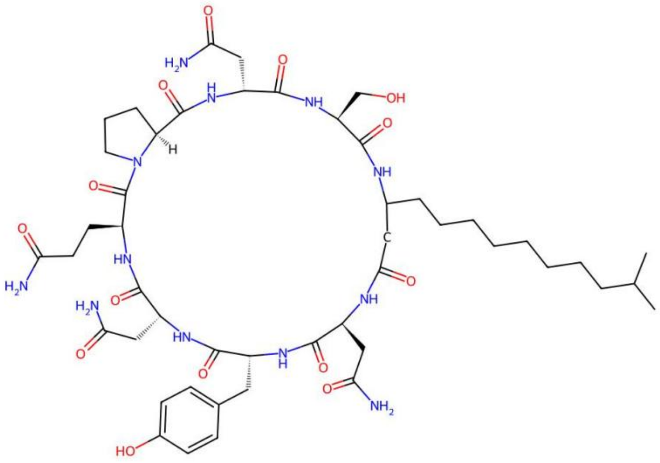

Figure S1. Chemical structure of iturin. The molecular structure of the cyclic lipopeptide iturin is schematically illustrated.

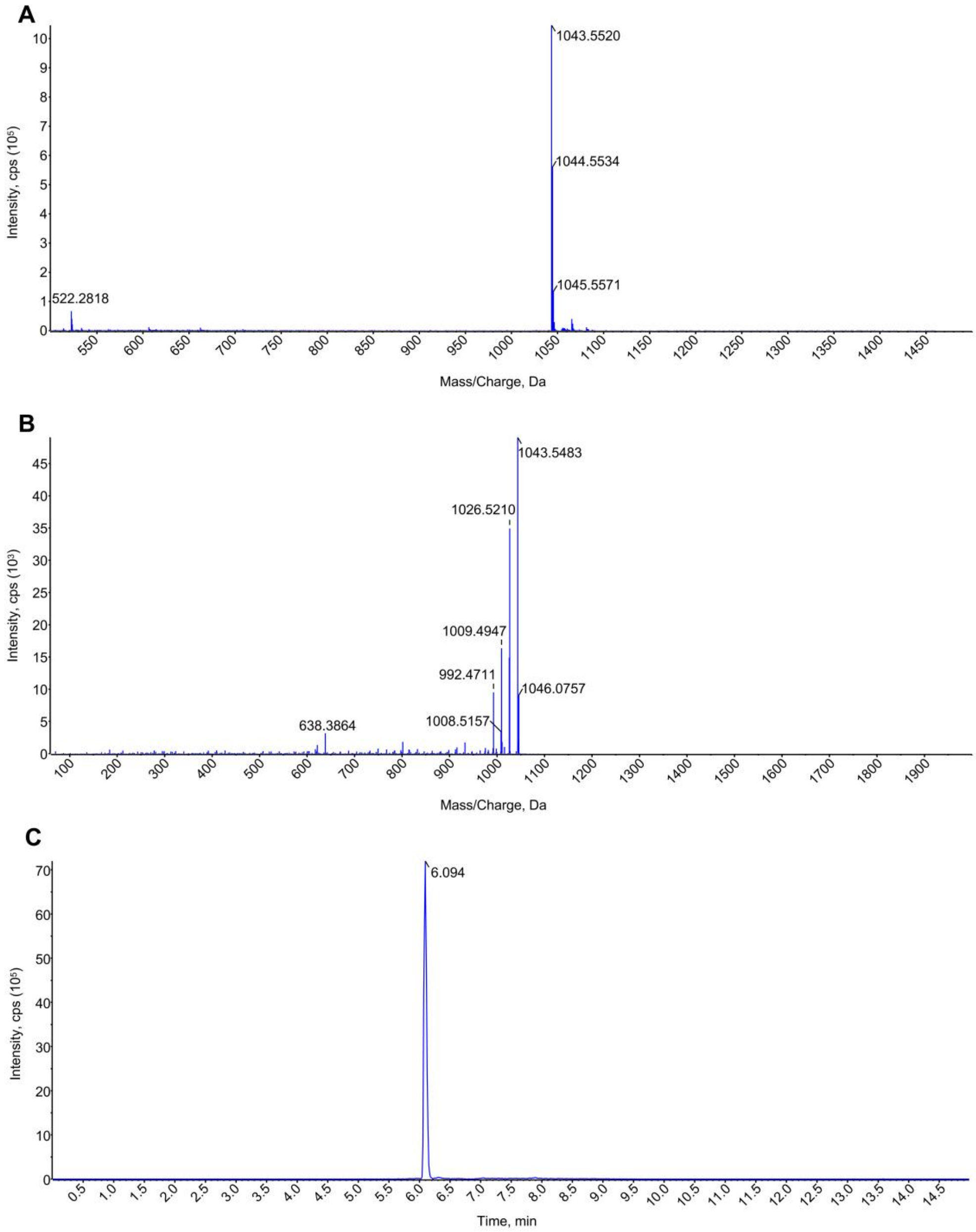

Figure S2. Mass spectrometric and chromatographic analysis of iturin.

(A) Mass spectrum (MS1) of purified iturin. The dominant peak at m/z 1043.5520 corresponds to the protonated molecule $[M+H]^+$.

(B) Tandem mass spectrum (MS2) of the precursor ion from (A), revealing characteristic fragment ions that confirm its cyclic lipopeptide structure.

(C) Chromatogram of the iturin sample, showing a single major peak at a retention time (t_r) of 6.094 minutes, indicating of high purity.

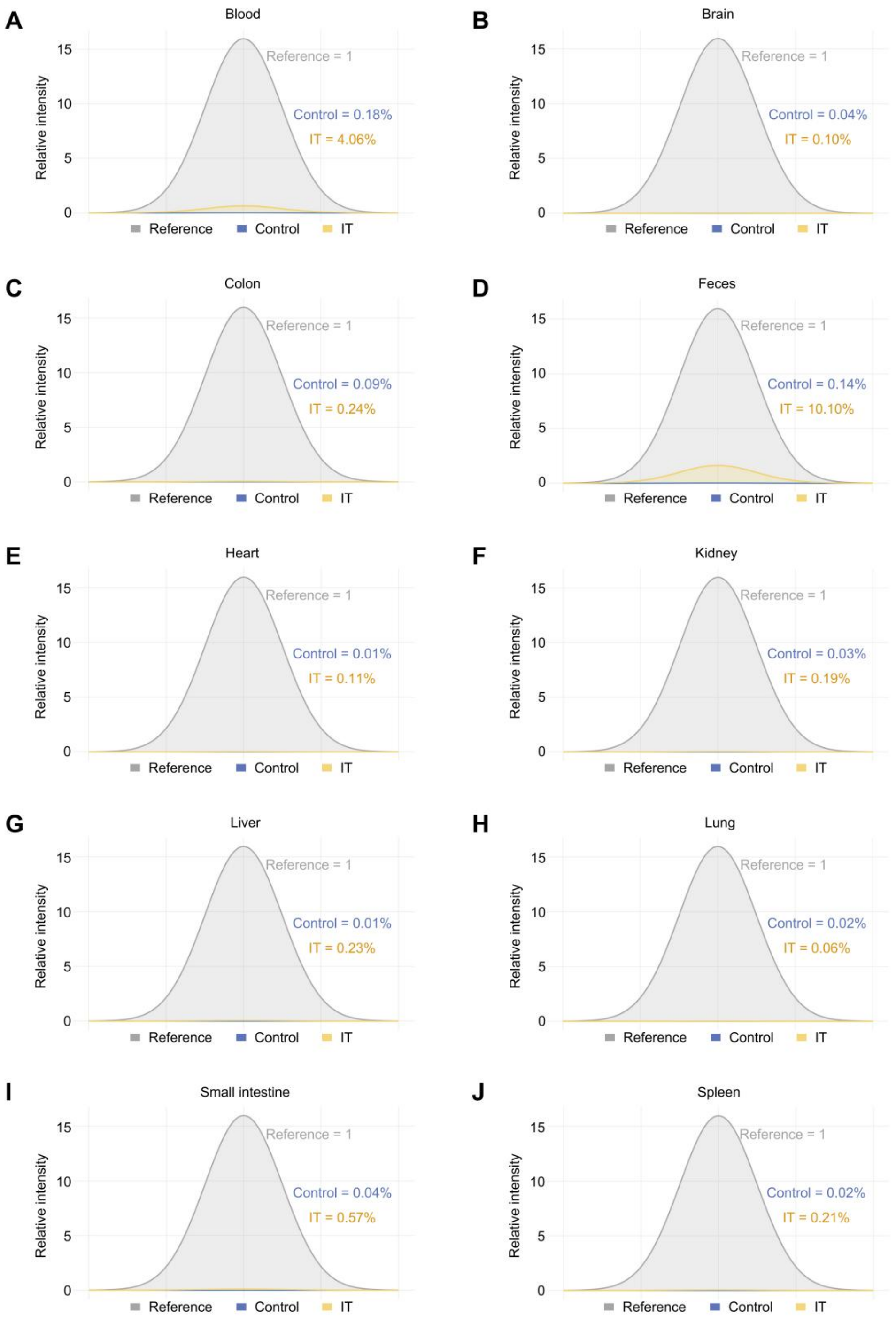

Figure S3. Tissue distribution of iturin in mice following a single oral administration.

(A-J) Relative levels of iturin in blood and various tissues 2 hours after oral gavage of IT (14 mg/kg) or vehicle control. IT levels were quantified by LC-MS/MS and are expressed as relative peak area ratios compared to a reference IT standard solution (2.1 mg/mL, equivalent to the concentration of the dosing solution administered). Panels show distribution in (A) blood, (B) brain, (C) colon, (D) feces (fecal content), (E) heart, (F) kidney, (G) liver, (H) lung, (I) small intestine, and (J) spleen.

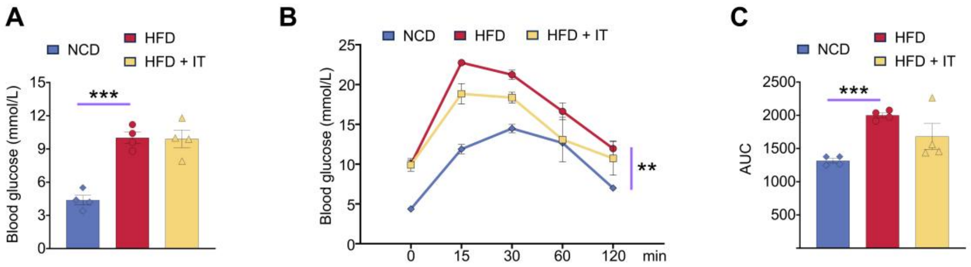

Figure S4. Oral glucose tolerance test (OGTT) in mice.

(A) Fasting blood glucose levels at baseline (0 minutes) (*n*=4 per group). Data are presented as mean ± SEM. Statistical significance was determined by unpaired Student's t-test, comparing NCD with HFD group, and HFD with HFD + IT group. ***$p < 0.001$.

(B) Blood glucose concentration profiles over time following an oral gavage of glucose (2 g/kg body weight) (*n*=4 per group). Data are presented as mean ± SEM. Statistical significance was determined by one-way ANOVA with Bonferroni post hoc correction. **$p < 0.01$.

(C) Quantification of the total glycemic response, represented as the area under the curve (AUC) for the data shown in panel (B). Data are presented as mean ± SEM. Statistical significance was determined by unpaired Student's t-test, comparing NCD with HFD group, and HFD with HFD + IT group. ***$p < 0.001$.

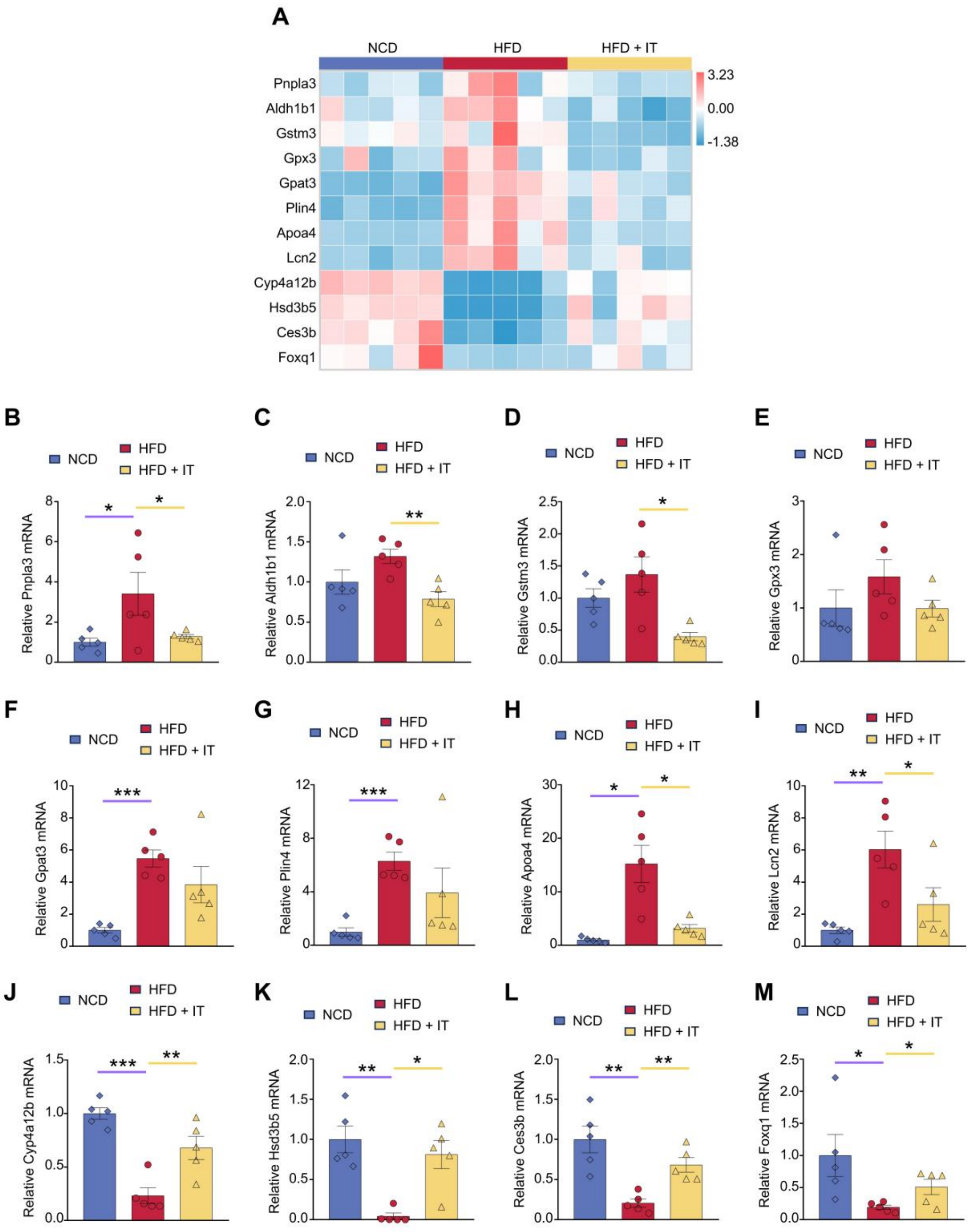

Figure S5. The verification of RNA-seq results through qRT-PCR analysis.

(A) The heatmap of the results of RNA-seq (*n*=5 per group).

(B-I) The mRNA level of downregulate genes in HFD-mice treated with iturin (*n*=5 per group). Data are presented as mean ± SEM. Statistical significance was determined by unpaired Student's t-test, comparing NCD with HFD group, and HFD with HFD + IT group. *$p < 0.05$, **$p < 0.01$, ***$p < 0.001$.

(J-M) The mRNA level of upregulate genes in HFD-mice treated with iturin (*n*=5 per group). Data are presented as mean ± SEM. Statistical significance was determined by unpaired Student's t-test, comparing NCD with HFD group, and HFD with HFD + IT group. *$p < 0.05$, **$p < 0.01$, ***$p < 0.001$.

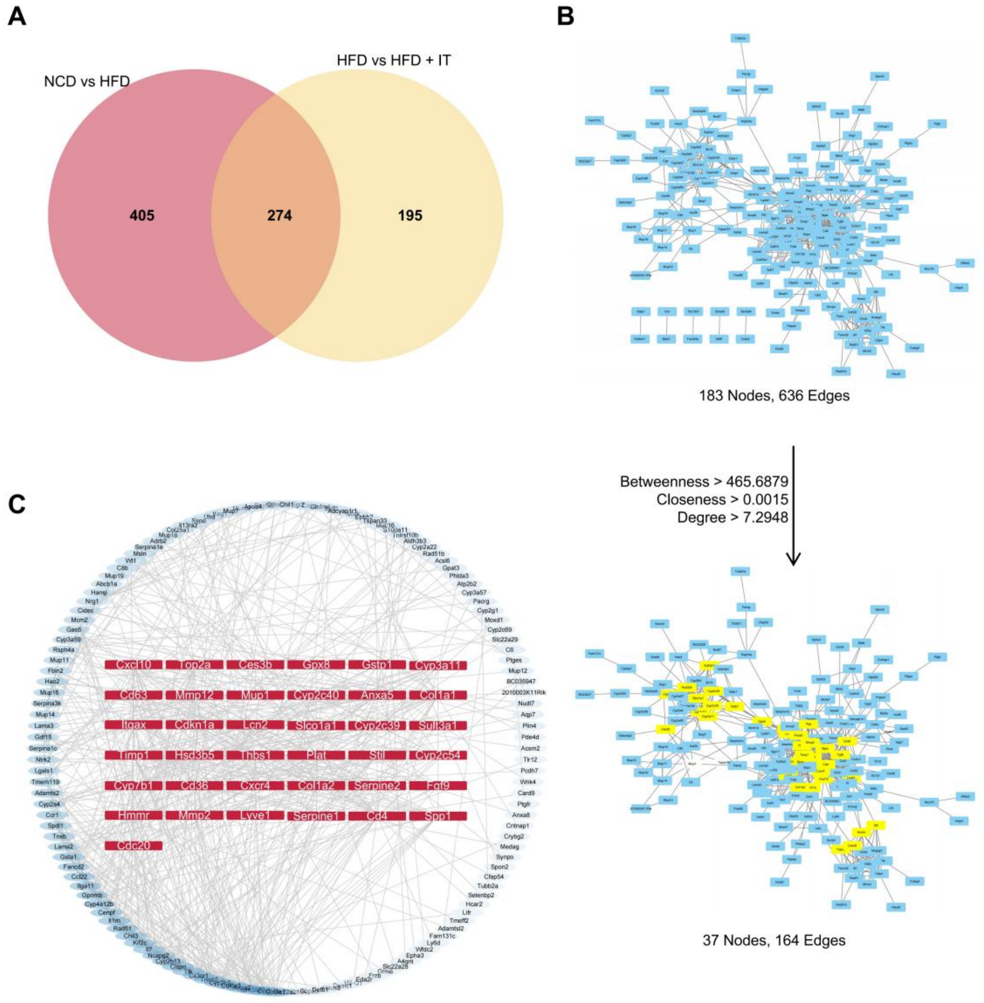

Figure S6. Central regulatory nodes of iturin analysed by Cytoscape.

(A) Venn diagram identifying overlapping and unique DEGs between the NCD vs HFD and HFD vs HFD+IT comparisons.

(B) The overlapping DEGs from (A) were used as the target set to construct a protein-protein interaction (PPI) network in Cytoscape, followed by topological analysis to score and rank nodes.

(C) Visualization of the PPI network, with the top-ranked central nodes highlighted, suggesting key regulatory targets of iturin.

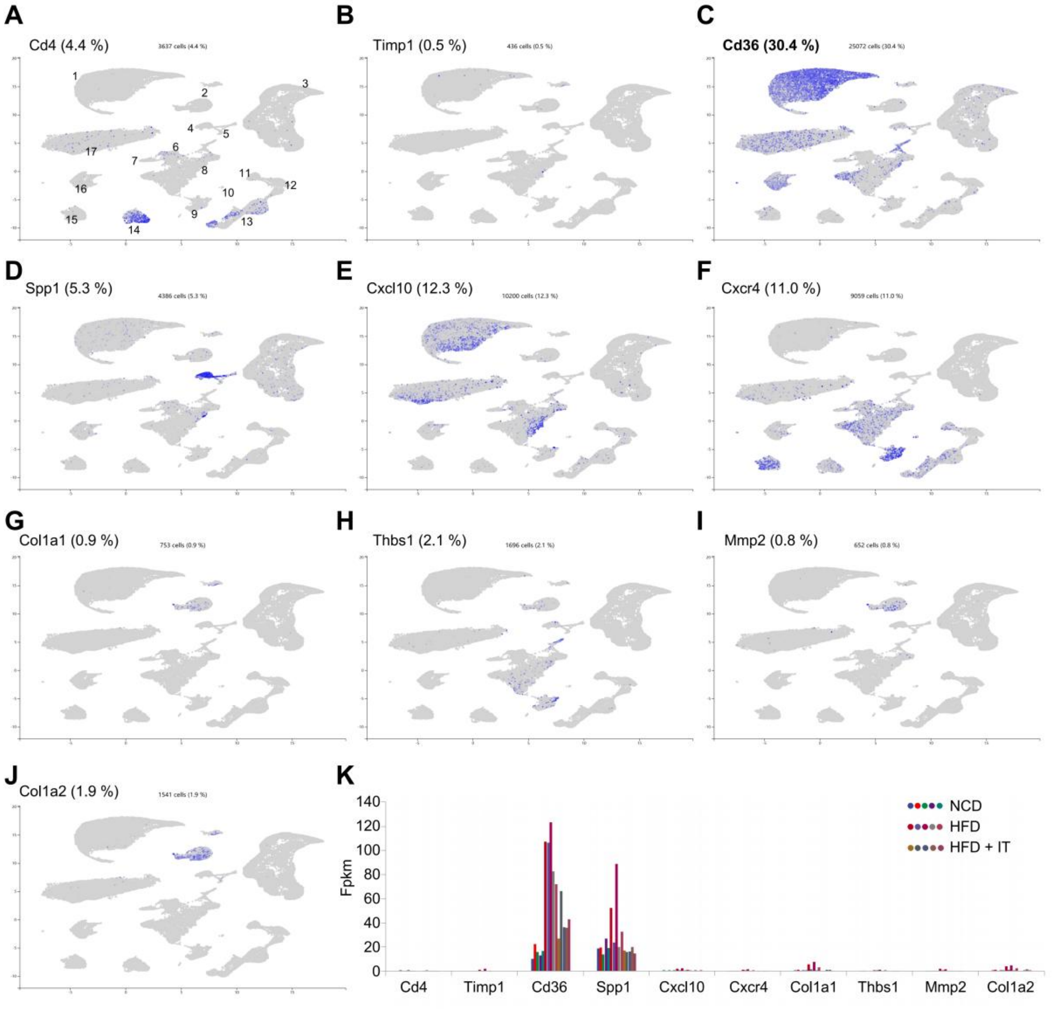

1. Endothelial cells; 2. Stromal cells; 3. Hepatocytes; 4. Cholangiocytes; 5. HsPCs; 6. cDC2s; 7. Mig cDCs; 8. Monocytes & Monocyte-derived cells; 9. Neutrophils; 10. Basophils; 11. NK cells; 12. ILC1s; 13. T cells; 14. pDCs; 15. B cells; 16. cDC1s; 17. Kupffer cells

Figure S7. The expression of top 10 nodes of iturin.

(A-J) The expression of genes in mouse liver cells (data were derived from the Liver Cell Atlas - https://www.livercellatlas.org).

(K) The fpkm of the top 10 central node genes under NCD, HFD, and HFD+IT conditions. Each colored bar represents data from an individual biological sample.

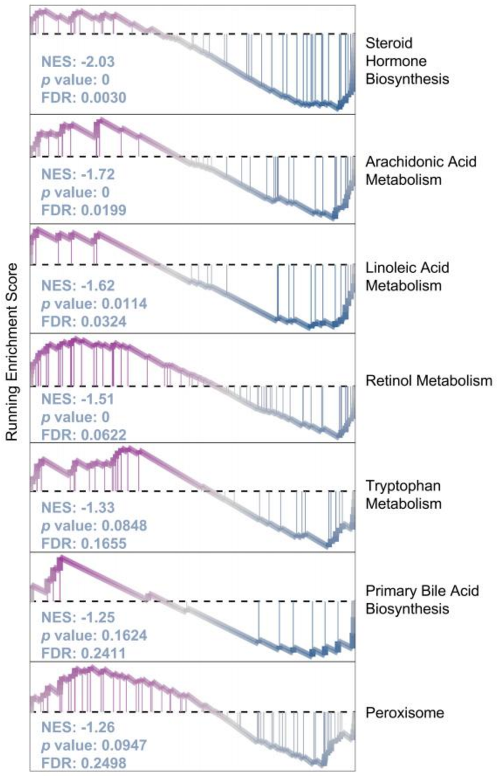

Figure S8. KEGG pathways negative correlation to *Cd36* (HFD + IT group) analysed by GSEA.

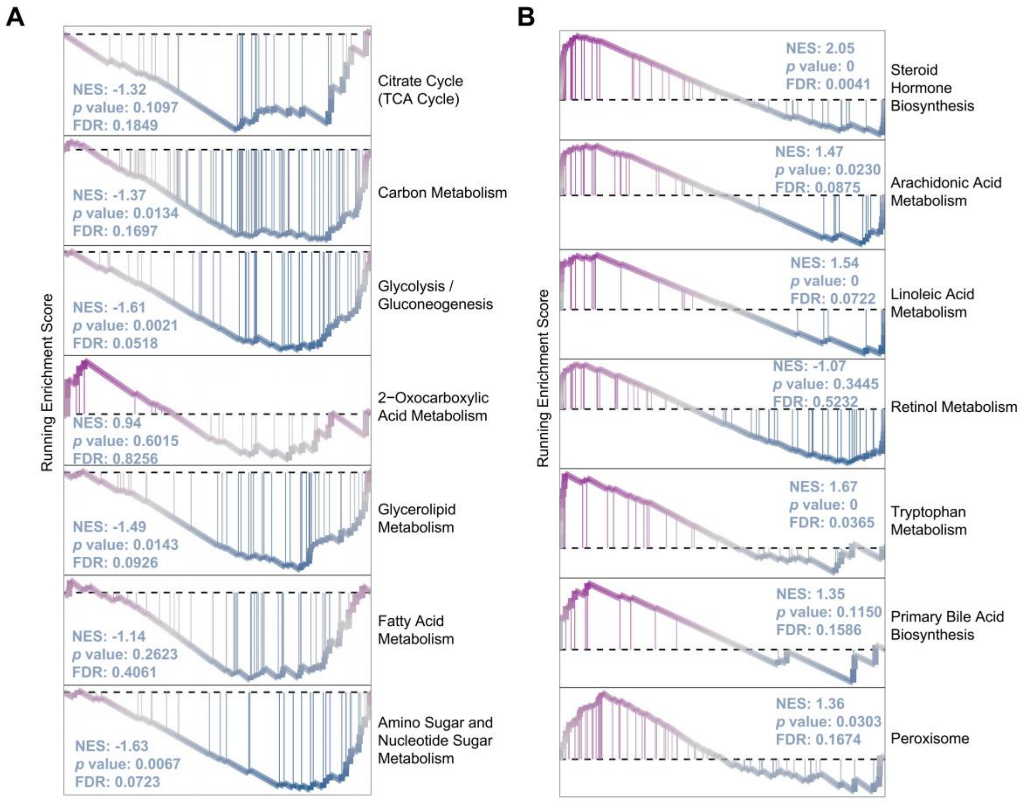

Figure S9. KEGG pathways following iturin supplementation analysed by GSEA (HFD + IT group).

(A) The downregulate pathways following iturin supplementation.

(B) The upregulate pathways following iturin supplementation.

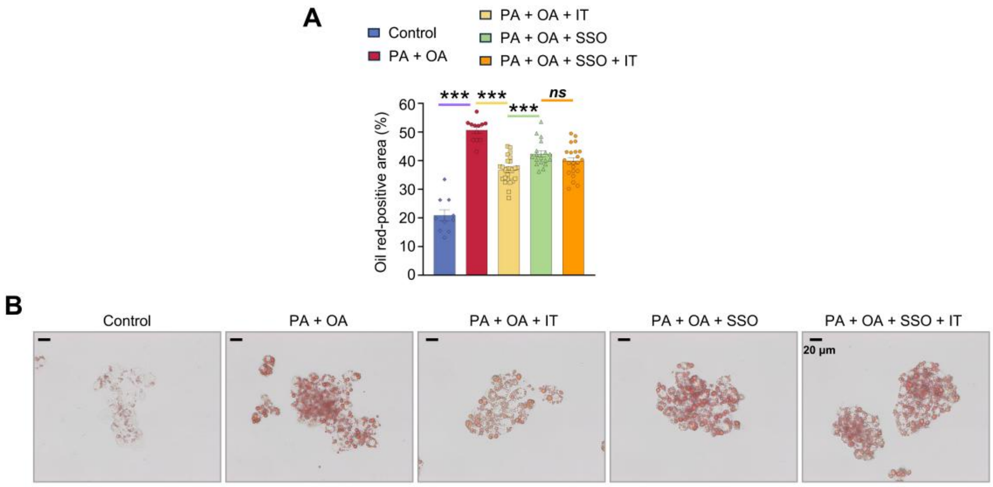

Figure S10. Iturin alleviates PA/OA-induced lipid accumulation in HepG2 cells, an effect dependent on Cd36.

HepG2 cells were treated with a mixture of 0.25 mM palmitic acid (PA) and 0.5 mM oleic acid (OA) for 24 hours to establish a cellular steatosis model. Cells were divided into five groups: Control (normal medium), PA + OA, PA + OA + IT (200 ng/mL), PA + OA + SSO (30 μM, a Cd36 inhibitor), and PA + OA + SSO + IT. Lipid accumulation was assessed by Oil Red O staining.

(A) Quantitative analysis of the Oil red O-positive area of cells. Data are presented as mean ± SEM. Each data point (*n*) represents the measurement from one cell, with cells pooled from three independent culture dishes per group ($n \geq 10$). Statistical significance was determined by unpaired Student's t-test for the following comparisons: Control vs PA + OA, PA + OA vs PA + OA + IT, PA + OA vs. PA + OA + SSO, PA + OA + IT vs PA + OA + SSO, and PA + OA + SSO vs PA + OA + SSO + IT. ***$p < 0.001$; *ns*, not significant.

(B) Representative images of Oil Red O staining.

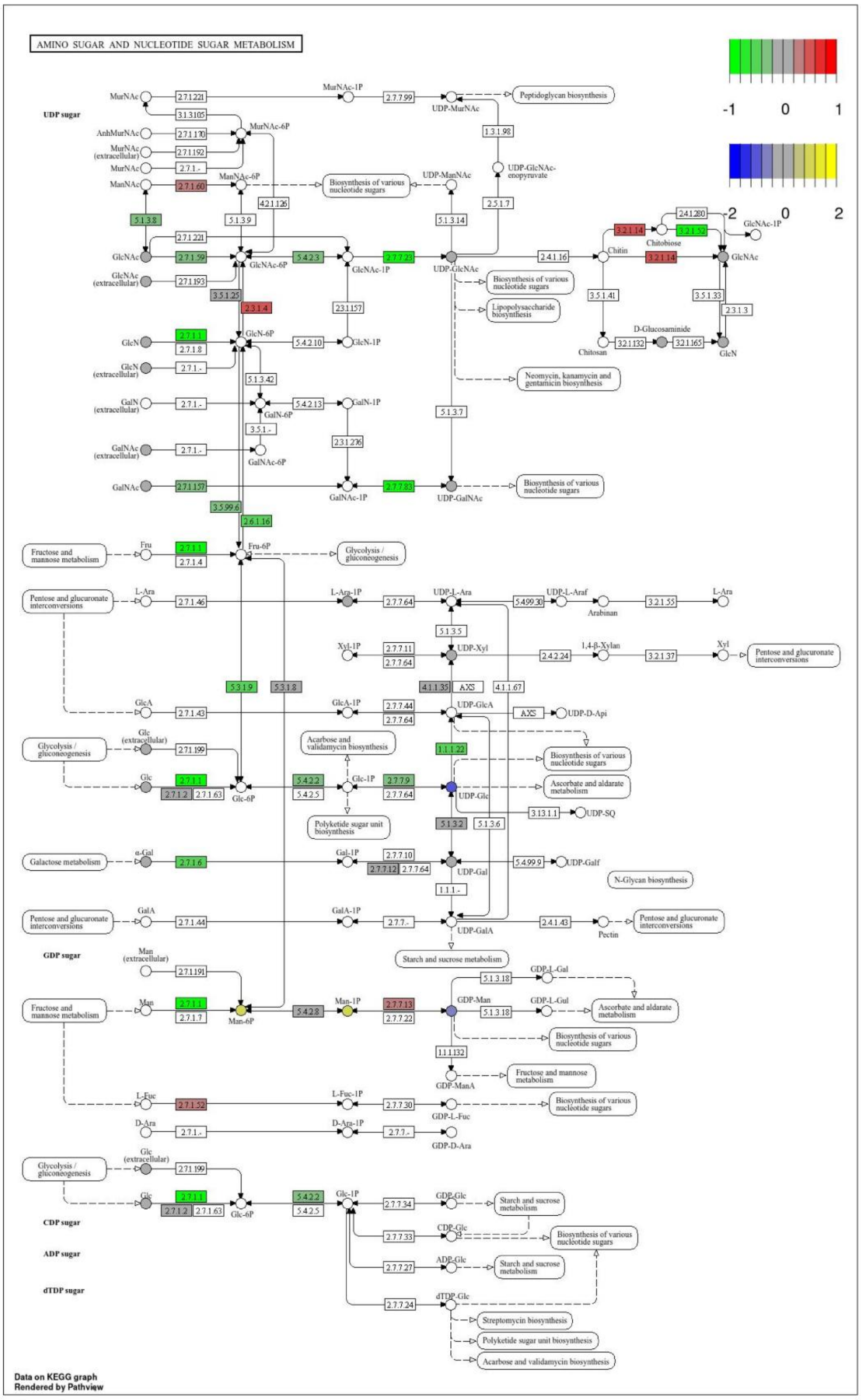

Figure S11. The amino sugar and nucleotide sugar metabolism pathway.

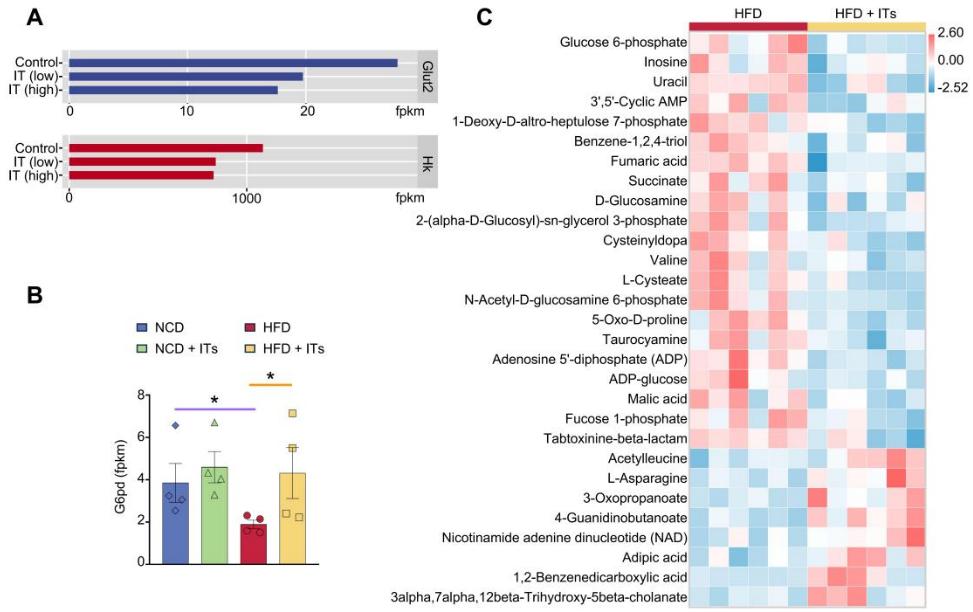

Figure S12. Iturin modulates glucose metabolism by inducing the upregulation of G6pd.

(A) The fpkm level of the Glut2 and Hk in HepG2 cells treated with iturin at a low (50 ng/mL) or high (200 ng/mL) concentration, compared to an untreated control group.

(B) The fpkm level of the G6pd (*n*=4 per group). Data are presented as mean ± SEM. Statistical significance was determined by unpaired Student's t-test, comparing NCD with HFD group, and HFD with HFD + IT group. *$p < 0.05$.

(C) The heatmap of the metabolites in livers of mice (*n*=6 per group).

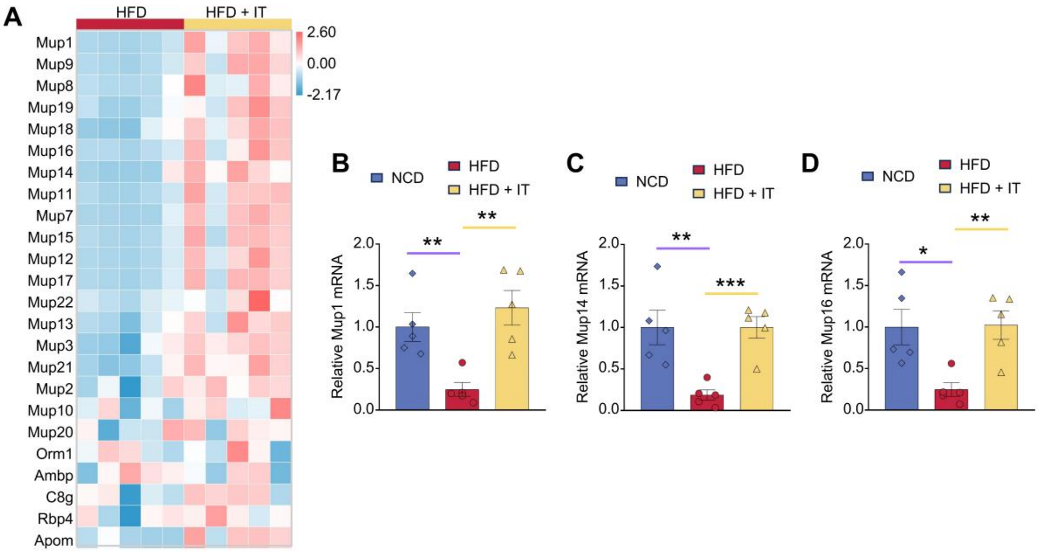

Figure S13. Alterations in the transcription levels of MUPs following the administration of iturin.

(A) The heatmap depicting the FPKM of lipocalin family (*n*=5 per group).

(B-D) The relative mRNA level of Mup1 (B), Mup14 (C) and Mup16 (D) (*n*=5 per group). Data are presented as mean ± SEM. Statistical significance was determined by unpaired Student's t-test, comparing NCD with HFD group, and HFD with HFD + IT group. **p* < 0.05, ***p* < 0.01, ****p* < 0.001.

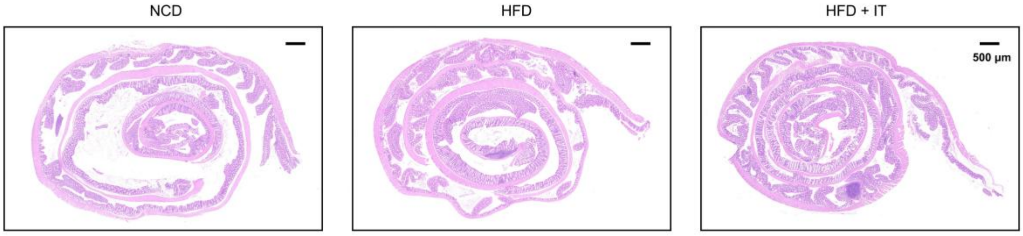

Figure S14. H&E staining of Swiss-rolled intestinal sections from mice.

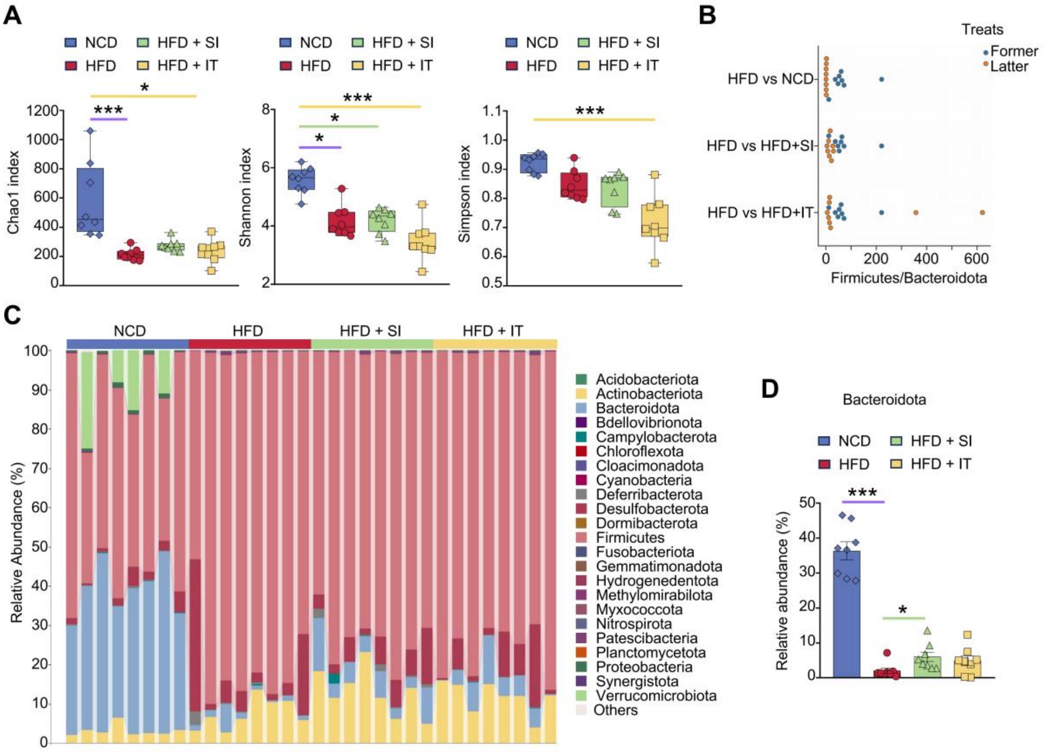

Figure S15. Impacts of iturin on intestinal microbiota.

(A) The alpha diversity index of the intestinal microbiota at the OTU level (*n*=8 per group). Data are presented as mean ± SEM. Statistical significance was determined by unpaired Student's t-test, comparing NCD with HFD group, NCD with HFD + SI group, and NCD with HFD + IT group. *$p < 0.05$, ***$p < 0.001$.

(B) The ratio of *Firmicutes* to *Bacteroidota* abundance (*n*=8 per group). Each dot represents an individual sample from the HFD (blue) or the NCD, HFD+SI, HFD+IT (yellow) groups.

(C) The phylum-level relative abundance of microbiota (*n*=8 per group).

(D) The relative abundance of Bacteroidota (*n*=8 per group). Data are presented as mean ± SEM. Statistical significance was determined by unpaired Student's t-test, comparing NCD with HFD group, HFD with HFD + SI group, and HFD with HFD + IT group. *$p < 0.05$, ***$p < 0.001$.

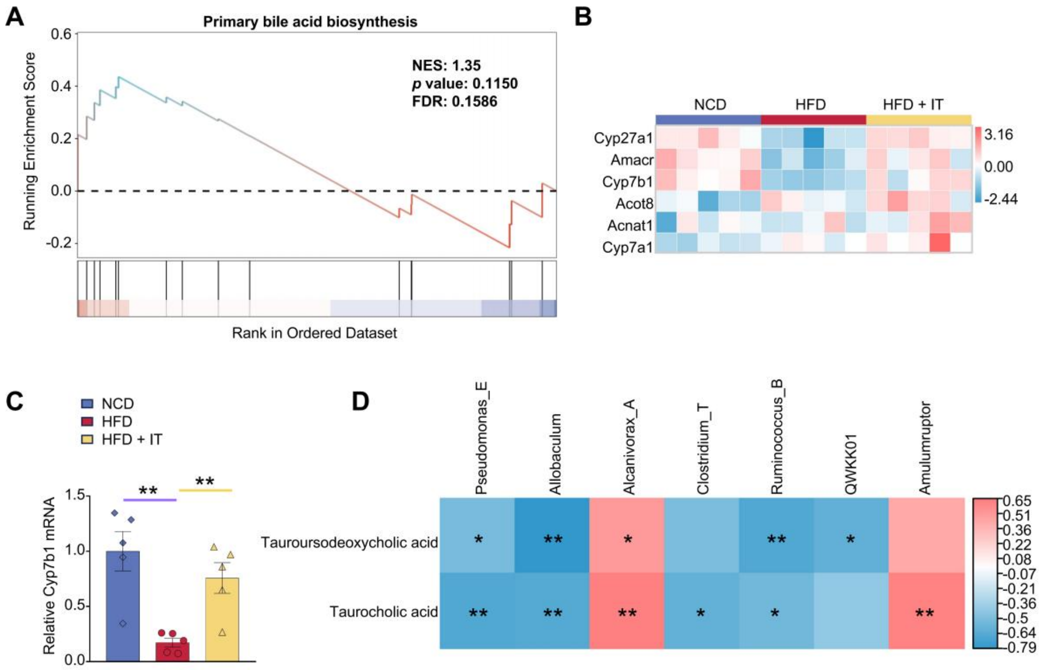

Figure S16. Impacts of iturin on primary bile acid biosynthesis.

(A) The primary bile acid biosynthesis pathway following iturin supplementation analysed by GSEA.

(B) The heatmap depicting the fpkm of leading edge genes in primary bile acid biosynthesis pathway (*n*=5 per group).

(C) The relative mRNA level of *Cyp7b1* tested by qRT-PCR (*n*=5 per group). Data are presented as mean ± SEM. Statistical significance was determined by unpaired Student's t-test, comparing NCD with HFD group, and HFD with HFD + IT group. **$p < 0.01$.

(D) *Spearman* correlation analysis assessing the relationship between the levels of taurosodeoxycholic acid, taurocholic acid, and microbiota abundance. *$p < 0.05$, **$p < 0.01$.

Table S1 Representative LC-MS chromatographic data used for the estimation of iturin purity via relative peak area normalization.

Peak No.	Retention time (min)	Peak area	Relative peak area (%)	Identity
**1**	**6.094**	**22,781,259**	**95.57**	**Iturin**
2	6.253	67,821	0.28	Impurity
3	6.317	242,351	1.02	Impurity
4	7.084	315,737	1.32	Impurity
5	7.799	72,122	0.30	Impurity
6	7.896	357,081	1.50	Impurity
Total	--	23,836,371	100	--

Table S2 Primer sequences of qRT-PCR for this paper.

Name	Sequence (5'-3')
Mup1-F	GAATGGCATACTATTATCCT
Mup1-R	GCACTCTTCATCTCTTAC
Mup16-F	CATACTATTATCCTGGCTTCT
Mup16-R	GCACTCTTCATCTCTTACA
Mup14-F	TCCATACTGTAGATGAAGAG
Mup14-R	ATGAGATGAGCCATAAGAA
Cd36-F	GAACAGAGGATGACAACT
Cd36-R	GGAACATAGAAGACTTGGA
Plin4-F	GTATTCATAAGAACACAGACA
Plin4-R	CATTGGTGGCTACATTAC
Lcn2-F	ATTACCCTGTATGGAAGA

Lcn2-R	AGAGAAGATGATGTTGTC
Cyp7b1-F	ATAGAAGATGGTAAGAAGAA
Cyp7b1-R	AAGCATAATCAGCAGTAA
Gstm3-F	TATGAGGAGAAGAGATATG
Gstm3-R	AATTAAGTAGGGCAGATT
Gpx3-F	CCAGTCTCAAGTATGTTC
Gpx3-R	GTTCTTCAGGAAAGTGTA
Foxq1-F	TACTCCTACATCGCTCTC
Foxq1-R	ATGAGGTACTCGTTGATC
Pnpla3-F	GCCTATGCTTTACAGATG
Pnpla3-R	TTAATCGCTTCACTCAGA
Gpat3-F	ATTACCATAACAAGCAGTA
Gpat3-R	CAGGATTAGGACATCAAT
Ces3b-F	CGATGAATACCTAGACAAT
Ces3b-R	GGAATGATGAACGAGATA
Aldh1b1-F	CTGGAGACGCTAGATAAC
Aldh1b1-R	TAACGGTACACCTTGATG
Hsd3b5-F	GACTCCCTTTCATCTATG
Hsd3b5-R	TTATGCTGTTGTTCTTCA
Cyp4a12b-F	CTATATCCAGGCAGTTGA
Cyp4a12b-R	GGACACTCTGTAGATGAT
Apoa4-F	AAGGAGAATGTGGACAAC

Apoa4-R	GCTCTTCCATATTCCTGTTA
Actinb-F	TATGGAATCCTGTGGCATC
Actinb-R	GTGTTGGCATAGAGGTCTT
